# TOAST: improving reference-free cell composition estimation by cross-cell type differential analysis

**DOI:** 10.1186/s13059-019-1778-0

**Published:** 2019-09-04

**Authors:** Ziyi Li, Hao Wu

**Affiliations:** Department of Biostatistics and Bioinformatics, Rollins School of Public Health, Emory University, 1518 Clifton Road NE, Atlanta, 30322 GA USA

**Keywords:** Reference-free deconvolution, Tissue-heterogeneity, DNA methylation, Gene expression, Cell-type composition

## Abstract

In the analysis of high-throughput data from complex samples, cell composition is an important factor that needs to be accounted for. Except for a limited number of tissues with known pure cell type profiles, a majority of genomics and epigenetics data relies on the “reference-free deconvolution” methods to estimate cell composition. We develop a novel computational method to improve reference-free deconvolution, which iteratively searches for cell type-specific features and performs composition estimation. Simulation studies and applications to six real datasets including both DNA methylation and gene expression data demonstrate favorable performance of the proposed method. TOAST is available at https://bioconductor.org/packages/TOAST.

**Electronic supplementary material:**

The online version of this article (10.1186/s13059-019-1778-0) contains supplementary material, which is available to authorized users.

## Background

There have been an increasing number of large-scale clinical studies using high-throughput technologies to profile biological samples collected from human subjects, in order to identify molecular biomarkers and therapeutic targets for different diseases [1, 2]. These samples (e.g., blood, tumor, or brain tissues) are often mixtures of different cell types. The importance of accounting for cell composition in high-throughput data analyses has been well-recognized [3–5]. For example, researchers proposed to include the compositions in regression models as covariates to adjust for the association between proportions and phenotype [6, 7], or to use them as inputs to solve for cell type-specific profiles [8]. Adjusting for cell composition is especially emphasized in epigenome-wide association studies (EWAS), where ignoring the composition has been shown to produce biased results [4]. As a result, adjusting for cell composition has become a standard procedure in EWAS studies [6, 9–11].

Regardless of the approach and goal, an important first step in the analysis of high-throughput data from complex tissues is to estimate the cell compositions. Experimental approaches including different cell sorting techniques [12, 13] are accurate, but too laborious and expensive to be used in large-scale studies. A number of computational methods based on signal deconvolution algorithms have been proposed. These methods mainly fall into two major categories: reference-based (RB) [14–17] and reference-free (RF) deconvolution [18–23].

There have been some discussions and comparisons of RB and RF deconvolution methods. It was reported that the RB deconvolution in general provides more accurate and robust estimation than RF deconvolution [17, 24, 25]. However, the application of RB methods are limited because it requires reference panels—the data from purified cell types. Currently, such reference panels only exist for a few tissue types, including blood [14, 26, 27], brain [6], and pancreas [28]. When reference panels are unavailable, for example in under-studied tissues or new high-throughput data modalities, RF deconvolution is the only viable solution. Moreover, the reference data are collected from a small number of samples with limited clinical conditions (mostly healthy subjects) and phenotypes such as age and gender. It has been reported that reference-based method fails to provide accurate cell composition estimation when the subjects of mixed tissues and reference panels have significant differences in clinical conditions and phenotypes, for example, when mixed samples are collected from newborns while the pure tissue samples are from adults [29]. In this case, reference-free deconvolution could be a better option [23, 30]. Due to these reasons, RF deconvolution is widely applied in recent studies of complex tissues [10, 31, 32]; therefore, new techniques with potential of improving RF deconvolution is worthy of further investigation.

The high-throughput data from complex samples are weighted averages of signals from different cell types. To solve for cell compositions, most RF deconvolution methods are based on some type of factor analysis. They usually apply on the data for a subset of “informative” features, or the ones containing information for the cell composition. It has been reported that feature selection plays an important role in the deconvolution and has great impacts on the accuracy of cell composition estimation [33–35]. Intuitively, the good features are the cell type-specific ones, i.e., the ones with distinct profiles in different cell types [36]. However, without reference panels, these features cannot be easily identified. As a result, most popular RF methods resort to ad hoc feature selection procedure. The variability of features has been commonly used as indicator of how “informative" a feature is for sample mixing [35, 37–39]. Using the most variable features in reference-free deconvolution is also recommended by a number of existing reference-free deconvolution publications [22, 23, 36]. A review of published studies that used RF deconvolution (Table 1) reveals that 8 out of 10 methods select the most variable sites as features.

**Table 1:** Summary of different feature selection techniques used by reference-free deconvolution methods in published studies

Features selected by	RF methods	Published studies
	Deconf	Liebner et al. [40]
	RefFreeEWAS	Johnson et al. [9]
	RefFreeEWAS	Johnson et al. [10]
Largest variability	RefFreeEWAS	Chen et al. [11]
	RefFreeEWAS	Everson et al. [41]
	ReFACTor	Kaushal et al. [42]
	ReFACTor	Rahmani et al. [23]
	NMF	Feng et al. [32]
External information	Deconf	Gaujoux et al. [43]
	RefFreeEWAS	Gasparoni et al. [44]

RefFreeEWAS is EWAS using Reference-Free DNA Methylation Mixture Deconvolution, from CRAN package *RefFreeEWAS*. ReFACTor is reference-free adjustment for cell type composition, from the *GLINT* package. NMF is non-negative matrix factorization, available from https://github.com/haoharryfeng/cfDNAmethy. Deconf is the in-silico deconfounding approach, i.e., alternate least-square NMF method using heuristic constraints, available from the *CellMix* package

In this work, we develop a straightforward and effective algorithm to improve RF deconvolution by better selecting features. The key idea is to identify features showing distinct profiles among different cell types, without knowing the pure cell type profiles or mixing proportions a priori. The feature selection procedure is purely data-driven, without requiring any additional information. The algorithm is based on a recently developed statistical framework, which provides functionality to detect cross-cell type differential signals for high-throughput data from mixed samples [45]. The proposed algorithm in this work iteratively performs feature selection (based on cross cell type differential analysis) and RF deconvolution and only needs a small number of iterations (less than 30) to achieve the best estimation.

We evaluate our method through extensive simulation and analyses of six real datasets and show that the proposed method can significantly improve the accuracy of proportion estimation based on existing RF deconvolution techniques. From our method, we observe substantial improvement in correlation and reduction in bias in the estimated proportions. In addition, there are significant improvements by using several other metrics including root mean squared error and goodness-of-fit of the deconvolution model, and precision of the selected features. Our method is applicable to both gene expression data and DNA methylation data as demonstrated in both simulation and real data applications. The method is implemented in the R package TOAST (TOols for the Analysis of heterogeneouS Tissues), which is freely available on Bioconductor (https://bioconductor.org/packages/TOAST).

## Results

### Method overview

**Fig. 1:**
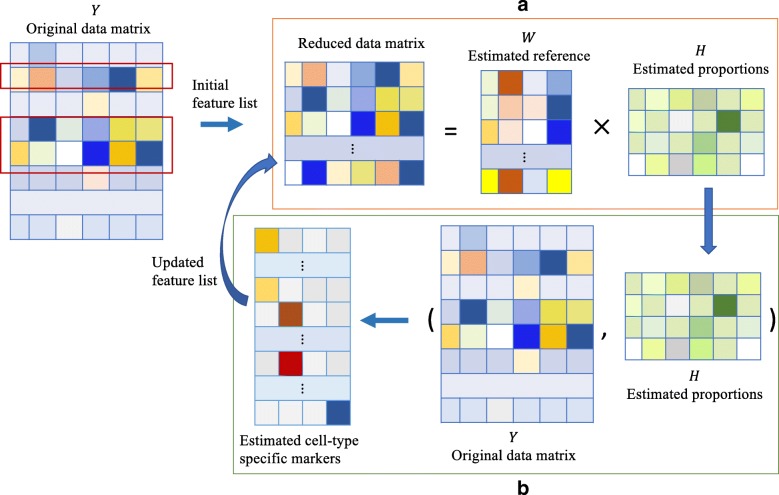
A schematic plot to illustrate the proposed method. We assume four cell types (*K* = 4) and six subjects (*N* = 6). Our method starts with the original data matrix **Y** and a list of initial markers. In step a, mixture proportions are estimated using existing RF deconvolution algorithms. In step b, cross-cell type differential analysis is performed to identify cell type specific-features as updated feature list. We improve the feature selection and RF deconvolution through iterating steps (a) and (b)

In this work, we develop an iterative algorithm to improve feature selection. Figure 1 summarizes the general workflow of the proposed method in an intuitive way. Given the original data matrix **Y** and a list of initial features, step (a) is to conduct an RF deconvolution to estimate mixture proportions. With estimated proportions, step (b) is to identify cell type-specific features using cross-cell type differential analysis. These features are then used for the RF deconvolution in step (a) in a new iteration. By iterating steps (a) and (b), the updated feature list can better capture the cell type distinction and improve RF deconvolution compared with initial features that are usually selected by choosing the most variable ones. The detailed notations and algorithm are described in the “Method and material” section.

The proposed method TOAST is essentially a feature selection method and thus could be used with existing reference-free algorithms to improve RF deconvolution. In order to evaluate the utility and flexibility of TOAST, we conduct extensive simulation and real data analyses of both gene expression data and DNA methylation data. We apply TOAST with the most popular reference-free methods, *deconf* [19] for gene expression data and *RefFreeEWAS* [22] and *BayesCCE* [23] for DNA methylation data.

### Simulation

To comprehensively assess the proposed method, we design a series of simulation studies where we can manually control the cell mixing procedure and sample sizes. Simulation data are generated based on real microarray experiments, one for gene expression and the other one for DNA methylation. We evaluate the impact of several factors on the deconvolution results, including sample size, initial marker selection, endpoint selection, and number of cell types in the mixture. The simulation procedure is described in detail in the “Method and material” section. In each simulation setting, the results presented are summarized over 100 Monte Carlo datasets.

To obtain a fair assessment of the proposed method, we adopt a number of metrics including correlations and root mean squared bias (RMSBias) of estimated versus true proportions, overlaps with true cell type-specific features, goodness of fit, and root mean squared error (RMSE) of the fitted deconvolution model. These metrics quantify the deconvolution results from the quality of estimated proportions, precision of selected features, and goodness of the overall deconvolution model. These metrics have been used by several previous studies [17, 23, 27, 46]. The details of calculating these metrics are available in the “Method and material” section. Using these metrics, higher correlations with true proportions, more overlaps with cell type-specific markers, higher goodness of fit, smaller RMSBias, and RMSE indicate better deconvolution performance.

#### Benchmarking TOAST through simulation

**Fig. 2:**
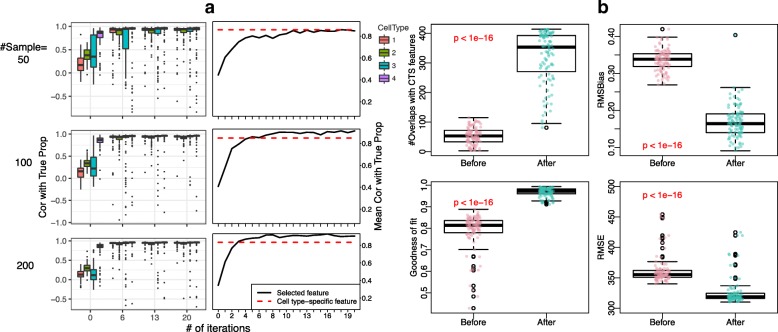
Results of the simulation study based on gene expression microarray dataset (GSE19830). **a** The correlations between estimated and true proportions by number of iterations. Left panel of **a**: boxplot of correlations for four cell types by number of iterations. Right panel of **a**: mean correlations across four cell types by number of iterations. **b** The number of overlaps with true cell type-specific (CTS) markers before and after iterations in the top left panel, the root mean squared bias (RMSBias) in the top right panel, the goodness of fit in the bottom left panel, and the root mean squared error (RMSE) in the bottom right panel. Sample size is 100 for **b**. *p* values in each panel are obtained using paired *t* test. Red font indicates being statistical significant. Top 1001–2000 most variable features are selected as initial features. Baseline performance is presented in the “number of iterations = 0” columns in **a** and “Before” columns in **b**

We first evaluate the proposed method in gene expression-based simulation, where *deconf* is used as the deconvolution method. Figure 2a shows the correlations of estimated and true proportions at initial point (number of iterations = 0) and after several iterations of applying the proposed method, for each of the four cell types (left panels) and averaged over four cell types (right panels). From the top row to bottom row, samples sizes increase from 50, 100 to 200. It is clear from left panels that overall the correlations between estimated and true proportions keep increasing during the iteration for all four cell types. The improvements are more dramatic and at the same time more stable with larger sample sizes. In the right panels, we compare the mean correlations over four cell types from the proposed method (black solid lines) versus by using the 1000 “real” cell type-specific features (red dashed lines). Here the real cell type-specific features are obtained from analyzing the pure cell type profiles. Note that these cell type-specific features are not available in real datasets. The results in right panels of Fig. 2a show that our iterative procedure achieves similar or even slightly better results than using true cell type-specific features. This indicates that our method is able to identify better cell type-specific features than using reference panels, which are usually of limited sample size (e.g., only one biological replicate in the current simulation study) and biological variances. In real world scenarios when reference panels are often obtained from a population different from the samples being studied, there could be biases in the reference. When the biases in the existing reference panels are large, TOAST can achieve better performance than using external reference panel.

Figure 2b shows the change of other metrics before and after applying the proposed method using 100 simulated samples. We observe that applying the proposed method significantly increases the number of overlaps with cell type-specific features (*p*<1*e*−16) and goodness of fit (*p*<1*e*−16) and significantly decreases RMSBias (*p*<1*e*−16) and RMSE (*p*<1*e*−16). Additional file 1: Figure S8 is a Venn diagram that intuitively presents the overlaps between initial, TOAST-selected, and cell type-specific features.

Additional file 1: Figure S1 presents the simulation results from the study based on DNA methylation data with *RefFreeEWAS*, one of the most popular reference-free deconvolution tools designed for DNA methylation data. We observe similar trend for the improvements in correlations with true proportions and in other metrics. Compared to Fig. 2, one major difference is that the application to DNA methylation data requires more iterations to converge, especially for smaller sample sizes. We suspect that it is related to the larger number of features in DNA methylation data (54,674 features in the simulated gene expression dataset versus 459,226 features in the simulated DNA methylation dataset), which leads to increased difficulty in identifying a good set of cross-cell type differential features. Nevertheless, these simulations demonstrate that the proposed method effectively improves the proportion estimation.

#### Initial feature selection

**Fig. 3:**
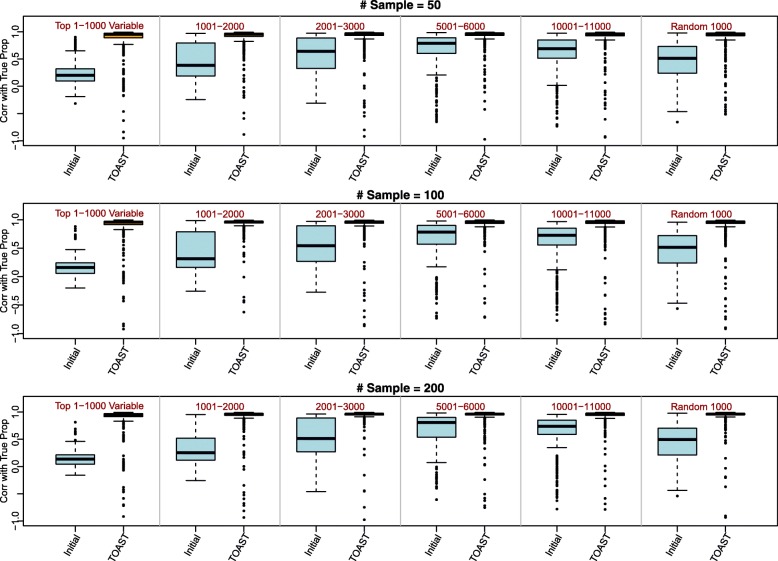
TOAST is stable with different initial feature selections in gene expression simulation studies. The panels from top to bottom correspond to sample sizes 50, 100, and 200. The panels from left to right correspond to different methods of selecting initial features: Top 1–1000 variables is to select the top 1000 most variable features, 1001–2000 is to select the top 1001–2000 most variable features, similarly 2001–3000, 5001–6000, 10001–11000 are to select the top 2001–3000, 5001–6000, 10001–11000 most variable features. Random 1000 is to randomly select 1000 features as initial features. In each panel, “Initial” and “TOAST” correspond to reference-free deconvolution results without and with TOAST. The presented results are summarized over 100 Monte Carlo experiments

As described in the “Background” section, variability is commonly used to select “informative” features for reference-free deconvolution. Along the same line, we consider six different approaches to select initial features, including the top 1000 most variable features; the top 1001 to 2000, 2001 to 3000, 5001 to 6000, and 10,001 to 11,000 most variable features; and 1000 randomly selected features. Figure 3 and Additional file 1: Figure S7 demonstrate that regardless of the method being used, the improvements from without applying TOAST (“Initial” columns) to after applying TOAST (“TOAST” columns) are consistent and stable across different sample sizes (rows) and data types (gene expression in Fig. 3 and DNA methylation in Additional file 1: Figure S7). Even a randomly selected set of initial features can lead to substantial improvements after applying TOAST (Additional file 1: Figure S4). This indicates that TOAST is very robust and stable to selections of initial features.

Closer comparisons of initial feature selections reveal that different initial features only have slight impacts on the converging rate. We compare using the top 1000 most variable features as initial features (Additional file 1: Figure S2, S3) versus using the second 1000 most variable features (Fig. 2, Additional file 1: Figure S1). We find the latter set of features requires fewer iterations to achieve a satisfactory deconvolution results than the former set. However, with enough number of iterations (e.g., 30 iterations), the ending correlations with true proportions are similar for these two sets of initial features.

To further investigate this phenomenon, we first check the overlaps between selected features and the true cell type-specific features (identified from pure profiles) in our simulation study with 100 simulated subjects. Additional file 1: Figure S6a shows an increasing trend for overlaps during the iteration. This trend is clear for both top 1000 and second 1000 most variable features as initial features, however, the second 1000 features have higher numbers of overlaps with cell type-specific features. This explains why selecting the second 1000 features could converge faster than the top 1000. With the increasing of iteration numbers, the difference between the two lines in Additional file 1: Figure S6a shrinks, which is consistent with the similar ending correlations by using the two sets of initial features.

In the same direction, we also compare the within-cell type standard deviations for different sets of features: the top 1000 most variable features, the second 1000 most variable features, and the features selected after applying TOAST, using the pure tissue profiles of the Mouse-Mix dataset (described later). As shown in Additional file 1: Figure S6b, the top 1000 most variable features have greater within-cell type variance than the second 1000 most variable features, and the features selected after applying TOAST have the smallest variation. This indicates that selecting the most variable features might not be a good idea in general, since the large variance can be from within-cell type (whereas one wants features with large between-cell type variation).

Considering the final performance using different initial features is similar, and the 1001–2000 most variable features have better converging rate than the top 1000 most variable features, we stick with using the 1001–2000 most variable features as initial features.

#### Ending point of iterations selection

**Fig. 4:**
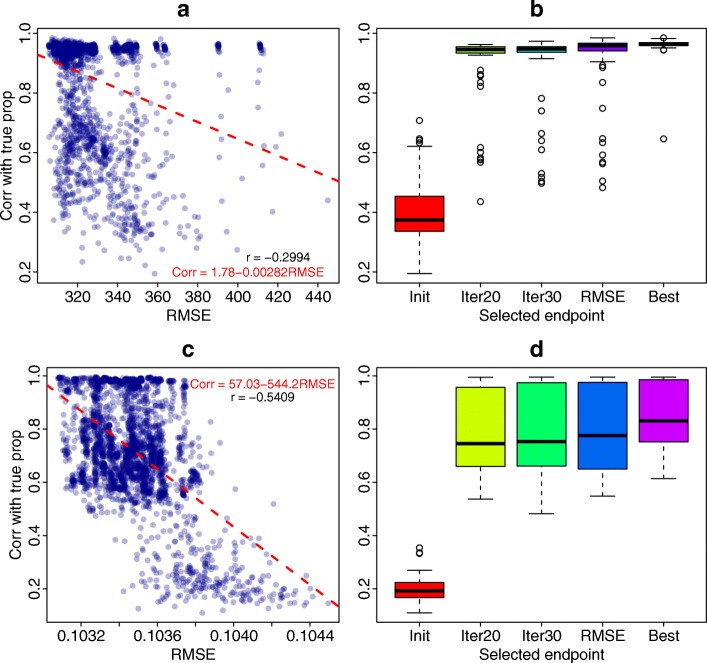
Exploration of endpoint selection. **a**, **b** Results from gene expression simulation settings. **c**, **d** DNA methylation simulation settings. **a**, **c** Negative correlations between RMSE and the correlations of estimated versus true proportions. **b**, **d** The boxplots of correlations with true proportion using different endpoint selection methods. Init is based on the initial features. Iter20 is based on features after 20 iterations. Iter30 is based on features after 30 iterations. RMSE is based on results with the smallest RMSE in 30 iterations. Best is the best possible correlations over all iterations (not obtainable in real data analysis)

In addition to initial feature selection, it is also important to understand how to choose the ending point of iterations. Ideally, we want to choose the iteration where the correlations of estimated and true proportions reach maximum. However, these correlations cannot be computed without knowing the true proportions. Root mean squared error (RMSE) of the fitted data from deconvolution methods has been used to choose tuning parameters in deconvolution algorithm [17]. Technically speaking, RMSE is not directly related to correlation with true proportions. However, RMSE reflects the fitness of the deconvolution model to the observed data, and better fitness usually leads to better proportion estimation. Figure 4a and c show the scatterplots of RMSE versus correlations with true proportions, from the gene expression and DNA methylation simulation studies, respectively. Significant negative correlations can be observed between RMSE and correlations with true proportions, indicating that smaller RMSE is related with better proportion estimation.

Figure 4b and d are boxplots of correlations with true proportions using different endpoint in the algorithm, from gene expression and DNA methylation simulations. *Init* is the results of using conventional RF methods without applying the proposed method. *Iter20*, *Iter30*, and *RMSE* are different ways of selecting endpoint. *Best* is the best correlation results from the 30 iterations. Note that *Best* is not observed in real data analysis but is presented here to make us aware of the best possible results.

We find no matter which endpoint selection method is used, the increase of correlations with true proportions over initial point is dramatic, which demonstrates the stability of the proposed method. Among the three endpoint selection methods, choosing by smallest RMSE results in the highest mean correlations in both gene expression and DNA methylation simulation studies. This finding also holds if we use randomly selected initial features (Additional file 1: Figure S5). Together with the significant negative correlations observed in Fig. 4a and c, we decide to choose the ending point of the proposed algorithm by the smallest RMSE. In our software, users could specify the total number of iterations, and among them, the iteration with the smallest RMSE would be chosen. Based on our experience, 30 iterations are sufficient for gene expression and DNA methylation datasets with four cell types and moderate or large sample size. The number of iterations should be increased for studies with smaller sample size (e.g., less than 50) or more cell types (e.g., 6 or more).

#### Impact of number of cell types in the mixture

For RF deconvolution, selecting an appropriate number of cell types is a difficult question. We provide more discussion toward the selection of cell type numbers in the “Discussions” section. Here we use our DNA methylation simulation study to explore the impact of having 6 cell types (CD4T, CD8T, Gran, Mono, NK, B cells) versus 4 cell types (CD4T+CD8T+NK, Gran, Mono, B cells) on RF deconvolution and the proposed method. As expected, increasing number of cell types leads to lower correlations of estimated proportions versus true proportions even after applying TOAST (Additional file 1: Figure S9).

We find increasing sample size is crucial for better proportion estimation, especially with more cell types. For example when there are 6 cell types in the mixture, correlations between true and estimated proportions from TOAST can be twice as high from 200 samples compared with 50 samples. Moreover, we find that TOAST provides greater performance improvement for 4 cell types than 6 cell types when sample size is moderate (50 or 100). This indicates that when sample size is small, it is better to specify a relatively small number of cell types and apply TOAST. If the experiment requires more cell types to be studied, increasing sample size is the most effective approach to improve deconvolution accuracy. In real data, the heterogeneous samples could contain many different cell types. However, the mixture is usually dominated by just a few cell types, so it is reasonable to just model the major ones. To what level the cell types should be combined and modeled is another question worth further investigations.

#### Compatibility with other RF method

TOAST is a feature selection method and works in conjunction with existing RF deconvolution methods for cell composition estimation. For all above results, TOAST uses *deconf* for gene expression and *RefFreeEWAS* for DNA methylation deconvolution. However, TOAST will work with other RF deconvolution methods and improve the results through better feature selection. Here we choose the state-of-the-art deconvolution method for DNA methylation data, BayesCCE [23], to demonstrate the flexibility of the proposed method. In their seminal paper published in 2018, BayesCCE has been shown to outperform existing deconvolution methods including ReFACTor [47], NNMF [20], and MeDeCom [48]. Here we compare performance of BayesCCE with and without applying TOAST for feature selection.

**Fig. 5:**
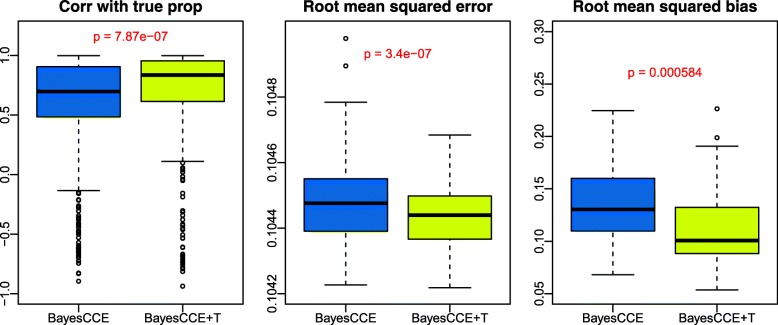
The proposed method could improve the deconvolution performance of BayesCCE in simulation studies. BayesCCE is the BayesCCE results without applying TOAST. BayesCCE + T is the BayesCCE algorithm with the proposed method TOAST. Results are based on 100 DNA methylation simulation datasets with four cell types. Panels from left to right demonstrate correlations of estimated versus true proportions (Corr with true prop), root mean squared error, and root means squared bias. 200 samples are simulated in each dataset. The *p* values are obtained from paired *t* test. Red fonts indicate significant test results. Boxplots are summarized from 100 Monte Carlo experiments

We find TOAST can significantly improve the deconvolution performance of BayesCCE (Fig. 5). Compared to BayesCCE, the proportions estimated by BayesCCE + T (BayesCCE with TOAST incorporated) achieve significantly higher correlation with true proportions (*p*=7.9*e*−07), smaller root mean squared error (*p*=3.4*e*−07), and root mean squared bias (*p*=5.8*e*−4). The improvement pattern holds in settings with different sample sizes (Additional file 1: Figure S10), and the improvement is more significant with the increase of sample size.

### Real data results

While simulation is useful to evaluate how well TOAST behaves in an idealized synthetic setting, simulation cannot inform us how well the deconvolution performs in reality. To fully evaluate TOAST in real world applications, we obtain six datasets including two gene expression, the Mouse-Mix data [8] and Immune data [14]), and four DNA methylation datasets, the EPIC (European Prospective Investigation into Cancer and Nutrition) data [49], Aging data [50], RA (Rheumatoid Arthritis) data [51], and Breast data [10].

These datasets are diverse in a number of aspects. For example, the sample sizes of these datasets range from 12 (Immune data) to 689 (RA data). Both Mouse-Mix and Immune data have true proportions from experiments, but the rest of the datasets do not have “true” proportions to provide benchmarks. As a surrogate, we obtain blood reference panels from [4] with profiles of six cell types (CD8T, CD4T, NK, Bcell, Mono, Gran) and apply RB deconvolution method *EpiDISH* [24] to obtain proportion estimates. Moreover, the Breast data is collected based on non-diseased breast tissue and does not have a reference panel to refer to. This is a perfect example to showcase the utility of RF deconvolution. To evaluate our method in the case with no prior information of cell type number of the tissue, we adopt goodness of fit in addition to correlation with true or RB proportions as metrics, as calculating goodness of fit does not require known true proportions (details demonstrated the “Method and material” section). The goodness of fit has been extensively used in previous studies to evaluate the deconvolution performance [27]. Even though it can be affected by the selected features to some degree, our simulation studies show that goodness of fit is highly and positively correlated with “correlations with true proportions” (Additional file 1: Figure S11). Thus, it is a reasonable metric to assess the deconvolution results since better goodness of fit is more likely to be associated with estimates having higher correlation with true proportions.

#### Benchmarking TOAST through six real data experiments

**Fig. 6:**
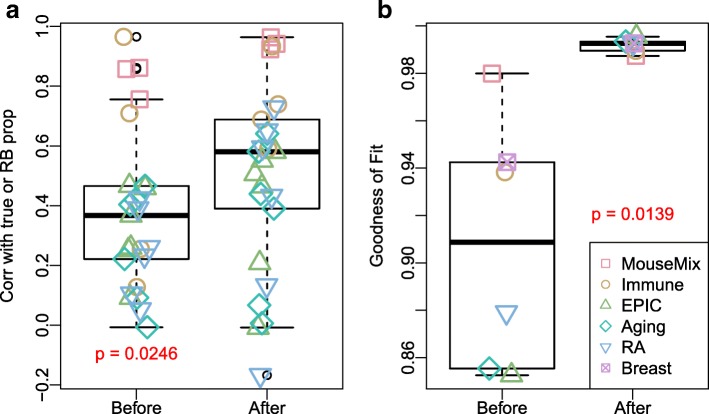
The proposed method improves the deconvolution results in real data applications. Boxplots show the evaluation metrics before and after updating features. **a** The correlation of estimated versus true (Mouse-Mix, Immune datasets) or reference-based deconvolution solved proportions (EPIC, Aging, RA datasets). **b** The goodness of fit for the deconvolution models before and after applying TOAST. The “Before” columns in both plots are baseline reference-free results without applying TOAST. Small jitter noises are added to points to provide better visualization

**Fig. 7:**
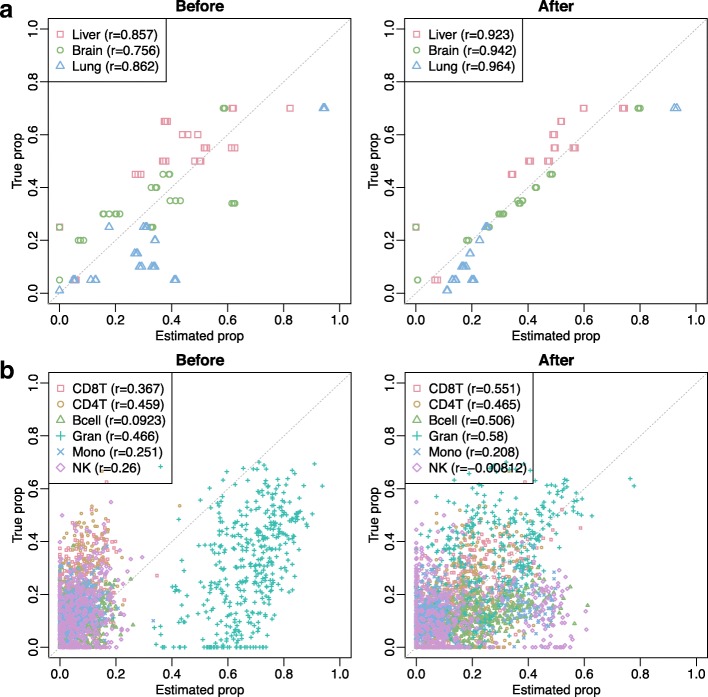
Proportion estimation from the Mouse-Mix dataset (GSE19830, upper panel) and EPIC dataset (GSE51032, lower panel). Left panels of **a** and **b**: estimated proportions versus true proportions without applying TOAST (baseline performance). Right panels of **a** and **b**: estimated versus true proportions after updating feature selection by the proposed algorithm. “True” proportion of the EPIC dataset is obtained using RB deconvolution method *EpiDISH*

Figure 6 summarizes the correlations and goodness of fit from all real data applications. These analyses reveal in most of cases a significant increase of correlations with true or RB proportions (*p*=0.0246) and in all cases a substantial increase of goodness of fit (*p*=0.0139). We further examine the proportions estimated before and after applying TOAST for each dataset. Figure 7 and Additional file 1: Figure S12–S14 shows the estimated versus true proportions at initial point (“Before”) and after applying TOAST (“After”). The improvements in proportion estimation can be dramatic for some datasets. For example in the application to the Mouse-Mix data shown in Fig. 7a, the correlations of estimated and true proportions for liver increase from 0.857 to 0.923 after applying the proposed algorithm. Similarly, the correlations increase from 0.756 to 0.942 for the brain and from 0.862 to 0.964 for the lung.

The improvements can also be observed in DNA methylation data applications. For example in the EPIC data experiment, the correlations increase from 0.0923 to 0.506 for B cells and from 0.367 to 0.551 for CD8T. It should be pointed out that, since we do not have true proportions in DNA methylation datasets, the results from RB deconvolution could itself be deviated from the truth. This might explain the correlations of these applications are lower than Fig.7a and Additional file 1: Figure S12, which have true proportions to benchmark the results. Nevertheless, our proposed method still demonstrates significant improvements in the composition estimation.

The Breast data have neither true proportions nor RB proportions because of the lack of pure tissue profiles; thus, we cannot present correlations nor scatterplot of proportions as with other datasets. We use the *RefFreeEWAS* package to determine the number of cell types, and six is selected in the analysis (consistent with [10]). The goodness of fit shows a dramatic increase before and after applying TOAST (Purple crossed squares in Fig. 6b).

We further demonstrate the compatibility of TOAST with BayesCCE and evaluate the performance in the three DNA methylation datasets (EPIC, Aging, and RA) which collect blood samples, as blood samples have prior knowledge of blood proportions provided in the BayesCCE paper. Additional file 1: Figure S16 shows that TOAST improves the performances from both RefFreeEWAS and BayesCCE. With TOAST, both methods have higher mean absolute correlations and lower root mean squared bias.

#### Comparison of RB and RF estimations in RA data

Finally, we ask whether the estimated proportions are biologically meaningful? Moreover, we are curious between RB and RF, which provides more meaningful estimation? Previous studies have shown that when reliable reference panel is available, RB deconvolution can obtain proportion estimates with high accuracy [14–17]. However, when the reference panel is obtained from subjects with different phenotypes such as age, gender, and disease status from the population of interests, RF could provide better proportion estimates than RB method [30].

RA dataset [51] epitomizes such a scenario, as it collects the whole blood from 354 RA patients and 335 normal controls with males and females in each group, while the blood reference panel is obtained from 6 healthy males in a separate study [26]. It was reported that RA can significantly change the proportions of some blood cell types in patients [52, 53], making blood cell proportions from RA patients differ from healthy subjects. Thus, the blood cell proportions can potentially be used for predicting RA. To compare the proportion estimations from different methods, we adopt a tenfold cross validation and use the estimated proportions to predict the disease status of each patient. Proportions that can better predict disease are deemed better estimated.

**Fig. 8:**
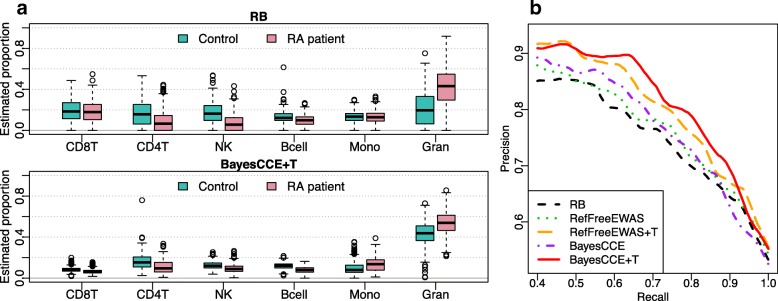
Results from the analysis of RA dataset (GSE42861). **a** The boxplots of estimated proportions of RA patients and controls from reference-based (RB) deconvolution method and BayesCCE with TOAST (BayesCCE+T). **b** The precision-recall curve for predicting disease status using estimated proportions from different methods. RefFreeEWAS and BayesCCE are baseline performance without applying TOAST. Results averaged from tenfold cross validation are used to generate the curves

We compare RB method EpiDISH, RF methods RefFreeEWAS, BayesCCE, and the RF methods with TOAST (RefFreeEWAS + T and BayesCCE + T) and summarize the results in Fig. 8. The left panel shows the proportions estimated from RB and BayesCCE + T, and the right panel shows the precision-recall curves for predicting RA from estimated proportions. Figure 8b shows that all RF methods achieve better disease prediction performance than RB method. Most importantly, TOAST can greatly improve the prediction performance of either RefFreeEWAS or BayesEWAS compared to the original methods, resulting in more biologically meaningful proportion estimation.

We also investigate the impacts of gender on the prediction performance (Additional file 1: Figure S15). In both male and female groups, we observe comparable or improvements after applying TOAST (yellow and red lines) over without TOAST (green and purple lines). These results again demonstrate the favorable and robust performance of the proposed method. On another note, we observe greater improvements by using TOAST in females than in males. In male populations, there is minimum advantage in reference-free deconvolution methods (colored lines) over reference-based method (black line), while the advantage is more profound in the female group and overall population. We believe this could be explained through gender distinctions in RA etiology [54–56] and sample size differences (197 males and 492 females). Nevertheless, TOAST still provides robust performance in improving proportion estimations and disease predictions for both male and female samples.

## Discussions

We present TOAST, a feature selection method for reference-free deconvolution to estimate cellular composition from high-throughput data of complex samples. We design an iterative algorithm, based on cross-cell type differential analysis, that improves feature selection and subsequently proportion estimation. Different from other methods that improve deconvolution performance through prior knowledge of markers or cell type proportions, TOAST is a purely data-driven method without requiring additional information. This provides great convenience for analyzing novel complex tissues or data from new modalities. TOAST can be incorporated with most, if not all, existing RF methods. The applications to deconf, RefFreeEWAS, and BayesCCE showcase this flexibility. If any prior information about cell type proportions are available, TOAST together with a RF method that utilizes such information, for example, BayesCCE, could further improve the estimation accuracy.

It is important to note that in general the RF deconvolution methods require large sample size to work well. As described in the “Background” section, sample deconvolution is important for analyzing data from large-scale clinical studies for human diseases. In such studies, large sample size is not only reasonable, but also necessary. In our opinion, the small sample size study is only reasonable for very homogeneous samples, such as cell lines or model organisms. To study heterogeneous and complex diseases in human subject, large sample size is necessary to provide enough power to identify disease biomarkers and therapeutic targets. This is the reason why most serious studies of human diseases have large sample sizes, for example, TCGA for cancers [57] and ROS/MAP for neurodegenerative diseases [58]. On another note, for datasets with small sample sizes, reference-free method is not recommended and one has to rely on reference-based method.

One universal difficulty of applying RF methods is to choose an appropriate number of cell types. Toward this end, we first want to join the discussions in previous publications and mention the usage of prior knowledge [23, 48]. For tissues that have been well-studied, such as blood and brain, prior knowledge about cell types can be easily obtained [26, 59]. When there is no prior information about the number of cell types, many RF methods provide schemes to select cell type number automatically, for example, by comparing the estimation error and the approximation error [48], or by AIC and BIC [20]. In our application to the Breast dataset, we use the “EstDimIC” function provided by the RefFreeEWAS package and choose six cell types, which is in consistent with previous analysis [10]. In addition, the selection of cell type numbers is dependent on sample size. As demonstrated in the simulation results, estimation accuracy is much lower for 6 cell types than 4 cell types with moderate sample sizes, even if the proposed method is applied. As a recommendation, when sample size is small and RF method is needed, one should consider to combine similar cell types and decrease the cell type number specified in RF deconvolution.

In the evaluations of initial feature selection, we consider six sets of selections, the top 1000, 1001 to 2000, 2001 to 3000, 5001 to 6000, and 10,001 to 11,000 most variable features and 1000 randomly selected features. These results provide comprehensive evaluations of the robustness of TOAST. Regardless of the initial features, TOAST could improve the feature selection and subsequently improve deconvolution accuracy.

The proposed method is primarily focused on microarray data (for gene expression or DNA methylation) in this work. However, the same principal is applicable for cell composition estimation in other data types, such as RNA-seq. Since the RF deconvolution method for RNA-seq is still underdeveloped, we did not test our functionality to deconvolve RNA-seq. Instead, we have evaluated our cross-cell type differential analysis method on RNA-seq data. We have designed a simulation study based on a real RNA-seq dataset [60] using Bioconductor package *PROPER* [61]. Detailed procedures of our RNA-seq simulation study have been described in Additional file 1: Section S2. As demonstrated in Additional file 1: Figure S17, TOAST is able to detect cross-cell type differential expressed genes (DEGs) with high accuracy (>70*%* of the top ranked 1000 genes are true DEGs), which means TOAST is able to accurately select desired features from RNA-seq data.

Our proposed method works in combinations with existing RF methods, such as deconf for gene expression data and RefFreeEWAS and BayesCCE for DNA methylation data. It is therefore important to follow the data preparation procedures suggested by those packages. For example, BayesCCE suggested to incorporate methylation-altering covariates into the analysis [23], which has been shown to generate more biologically meaningful results in our real data applications.

TOAST also demonstrates favorable computationally performance since the feature selection step is based on linear regression. We have benchmarked TOAST on a laptop computer with 4GB RAM and Intel Core i5 CPU. For a real gene expression dataset with 54,675 features and 100 samples, it takes less than 2 min to complete 30 iterations. For a real DNA methylation dataset with 459,226 features and 100 samples, it takes around 8 min to complete 30 iterations.

Finally, the proposed methods have some connections to SVA (Surrogate Variable Analysis) [62] and RUV (Remove Unwanted Variation) [63, 64]. Both RUV and SVA are targeting at the removal of “unwanted” variations or “undesired” confounding factors. For complex tissues, the cell compositions, which we are interested in, might be considered as unwanted variations under their context. All methods use some type of factor analysis (SVD for RUV and SVA, and NMF for TOAST), and the estimated “unwanted variations” (or proportions in our study) can be used for downstream analysis by including them as covariates in a linear model. One important distinction in TOAST is the iterative feature selection procedure, since SVA and RUV use a fixed set of features. From this perspective, an extension of SVA, the ISVA (Independent Surrogate Variable Analysis) [65], has more similarity to TOAST for its data-driven feature selection. However, ISVA identifies informative features through regressing observed data on each individual surrogate variable (SV). If the SVs contain proportion information, this approach can be considered as a special case of TOAST. Moreover, ISVA only performs one round of feature selection instead of iterating between feature selection and ICA (independent component analysis). This may not be ideal since we have shown that the iteration greatly improves the results.

## Conclusion

We study the problem of feature selection in RF deconvolution for cellular composition estimation from high-throughput data of complex samples. We design an iterative algorithm, based on cross-cell type differential analysis, that improves feature selection and subsequently proportion estimation. There are two advantages of the proposed methods. First, our algorithm is flexible enough to work with existing RF deconvolution methods. The applications to gene expression data and DNA methylation data showcase this flexibility. Second, our current results show that only a few iterations (e.g., 30 iterations) can achieve good improvements, which means it is computationally efficient. With the wide applications of RF deconvolution and the increasing needs of analyzing heterogeneous samples, we expect broad applications of the proposed method to microarray data and to other omics data as well.

## Method and material

### Notations and model

We first provide a formal definition of the problem that most RF deconvolution algorithms try to solve. Denote the data generated from high-throughput experiments by **Y**, a *P* by *N* matrix with rows representing features (genes, CpGs, etc.) and columns representing samples. Assume **Y** contains mixed signals from *K* (assumed known) “pure” cell types. Most deconvolution methods seek the optimal solutions for matrix factorization **Y**=**WH**. Here reference panel **W** is a *P* by *K* matrix, where the *k*-th column of **W** is the profile of cell type *k*. *H* is the mixture proportion matrix with dimension *K* by *N*, each column represents the mixture proportions of *K* cell types for each subject. **H** has a constraint that every column sums up to one. If the input is DNA methylation data, another constraint, in which elements of **W** are bounded by 0 and 1, is added to the algorithm. **H** is the variable of interests in this analysis.

Using this notation, selecting high variable features is equivalent to selecting rows with highest variance $Var(Y_{i\cdot }) = \sum _{j} (Y_{ij} - \bar {Y}_{i\cdot })^{2}$, which contains contributions from within-cell type variances (biological variation among samples for pure cell types), cross-cell type variances (mean differences among pure cell types), and variation from the mixing proportions. As discussed in recent published studies [32, 46], the good features for deconvolution are those with low within-cell type variation and high cross-cell type variation. If we select features solely based on variance of raw observations, features with high within-cell type variances could also be included, which will have negative impact on the RF deconvolution in later step.

Now we briefly introduce the method for cross-cell type differential analysis for data from mixed sample, which is a special case of our previously proposed method [45]. Assume the observed data for the *p*-th feature are ***Y***_***p***_=[*Y*_*p*1_,*Y*_*p*2_,⋯,*Y*_*pN*_]^*T*^, *p*=1,⋯,*P*. Denote the proportions obtained for sample *s* are ***θ***_***s***_=(*θ*_*s*1_,*θ*_*s*2_,⋯,*θ*_*sK*_). With known proportions, the observed data can be modeled by a linear model:

1 $$ E(\boldsymbol{Y_{p}}) = \boldsymbol{V}\boldsymbol{\beta_{p}}  $$

where

2 $$ \boldsymbol{V} = \left[\begin{array}{llll} \theta_{11} & \theta_{12} &\cdots & \theta_{1K} \\ \theta_{21} & \theta_{22} &\cdots & \theta_{2K} \\ \vdots & \vdots & & \vdots \\ \theta_{N1} & \theta_{N2} &\cdots & \theta_{NK} \\ \end{array}\right],~~~ \boldsymbol{\beta_{p}}= \left[\begin{array}{l} \mu_{p1} \\ \mu_{p2} \\ \vdots \\ \mu_{pK}\\ \end{array}\right].  $$

Regression coefficient *μ*_*pk*_ represents the mean level for the *p*-th feature in the *k*-th cell type. Using this model, one can test the difference between cell type *k* versus other cell types in feature *p* by following hypothesis test:

3 $$ H_{0}: \mu_{pk} - \frac{1}{K-1}\sum_{i\neq k} \mu_{pi} = 0, \quad k = 1, \cdots, K.  $$

Features with significant test results are cell type-specific features.

The above linear model can be fit using ordinary least squares (OLS) method. We find that sometimes extremely small variance estimation can lead to undesirable results for some features. To overcome this problem, we impose a data-driven lower bound (10th quantile values of all estimated variances) to stabilize the variance estimates. Similar methods are widely used in popular tools for differential expression analysis which has been proven to have good results [66]. In addition, as all the *μ*_*pi*_’s represent the mean observation levels for each cell type, it may not be reasonable to have negative estimators. As such, we provide options to bound negative estimated parameters in the TOAST software. In our experiments, we find bounding negative estimators has minimum impact on the results due to the small proportions of negative estimators (e.g., less than 2% in RA data analysis).

With this method, we design the following iterative algorithm to improve feature selection in RF deconvolution. The algorithm starts with a list of initial features, denoted as *M*_0_, which could be selected using conventional methods, such as choosing the top variable features. In the “Initial feature selection” section, we have provided more discussion about this. Observed data for these features, denoted as $\mathbf {Y_{M_{0}}}$, are used as inputs for RF deconvolution to estimate mixture proportions. With the estimated proportions, we run cross-cell type differential analysis on the whole observed data **Y** to detect cell type-specific features. In each iteration, the top features from this analysis with the same length as *M*_0_ are then used for another round of RF deconvolution. In our software implementation, the function “DEVarSelect()” has an argument “nMarker” for users to specify the number of initial features and selected features in each iteration. The default value is 1000, as used throughout this paper. The feature selection and RF deconvolution are iterated for a number of times then stop. In the “Ending point of iterations selection” section, we have more discussions about choosing endpoint. The algorithm is summarized below.


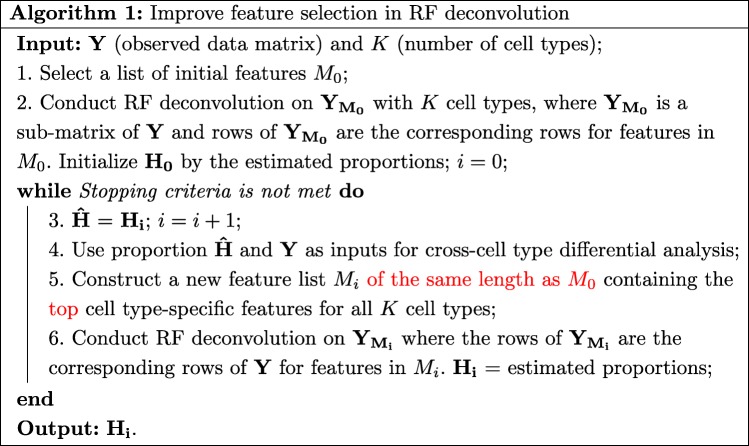


Note that Algorithm 1 is not constrained to a specific deconvolution method, so most existing RF methods can be applied in conjunction with this procedure. For example in our simulation study and real data applications, we use RF algorithm *deconf* [19] for gene expression microarray data and *RefFreeEWAS* [20] and *BayesCCE* [23] for DNA methylation microarray data.

The proposed algorithm guarantees the improvements of proportion estimations from two aspects. The first is that RF deconvolution performs better with more “correct” or informative features. The second is that feature selection can be improved with more accurate estimated proportions. The first point has been empirically demonstrated by all our results and by discussions about feature selection in many RF deconvolution publications [23, 36]. The latter point is a natural result from the measurement error model [67, 68].

### Simulation setting

We design two simulation studies based on real datasets, one for gene expression data and the other one for DNA methylation data, so that the simulation studies mimic the real data scenarios well.

The first step of our simulation studies is to generate subject-specific reference panels. In the first simulation study, the four cell types in the reference panels for each individual are simulated from log-normal distributions, with cell type-specific means and variances estimated based on a real gene expression microarray dataset obtained from the Gene Expression Omnibus (GEO) with accession number GSE11058 [14]. This dataset provides gene expression profiles of four purified immune cell line tissues and their manually mixed samples. We only use the data from four purified cell line tissues to estimate the cell type-specific mean and variance of each feature. In the second simulation study, the four cell types in the reference panels are simulated from normal distributions, with cell type-specific means and variances estimated from the GEO dataset GSE35069 [26]. Note that GSE35069 has DNA methylation measurements for six types of purified blood cells (CD4T, CD8T, B-cell, Mono, Gran, NK, Gran). For our simulation study, we combined CD4T, CD8T, and NK to one pseudo-cell type when estimating the cell type-specific mean and variance of each feature. In the “Impact of number of cell types in the mixture” section, we explore the impacts of using 6 cell types to deconvolve observed signals versus using 4 cell types.

After subject-specific reference panels are generated, the simulated pure cell types are manually mixed using known mixture proportions, which are simulated from a Dirichlet distribution with parameters (0.968,4.706,0.496,0.347) for four-cell type setting or (0.89,4.12,0.47,0.33,0.61,1.02) for six-cell type setting. Randomly simulated measurement errors are added to the mixed signals. For all settings, results are summarized over 100 Monte Carlo datasets.

### Evaluation metrics

We calculated a number of metrics to evaluate the proposed method. First, we compute the correlations and biases for comparison between estimated and true proportions. Correlations with true proportions is widely used in almost all studies that involve evaluating the deconvolution performance [14, 17, 20, 24]. Specifically, we calculate:

4 $$ Corr~with~true~prop = diag(r(\mathbf{H}, \hat{\mathbf{H}}))  $$

where *r*(·) represents the Pearson correlation and *diag*(·) is the diagonal operation extracting the diagonal elements from the variance-covariance matrix. RMSBias is the root mean squared bias of the estimated versus the true proportions, i.e.,

5 $$ RMSBias = \sqrt{\sum{(\mathbf{H} - \hat{\mathbf{H}})^{2}}/KN}.  $$

Number of overlaps between selected and cell type-specific markers is to evaluate the agreement between our selected markers, and the best markers can be chosen if pure tissue profiles are known. The cell type-specific markers are selected by the largest log fold changes of the cell type-specific value against the mean value of other cell types, iterating over all cell types (details presented in Additional file 1: Section S1). Higher overlap usually leads to better proportion estimation.

Goodness-of-fit score is the Pearson correlation of real observations and fitted values from the RF deconvolution results, which we find is a metric especially suitable when true proportions are unknown [27]. Briefly, for estimated basis matrix $\hat {\mathbf {W}}$ and cell proportions $\hat {\mathbf {H}}$, the reconstructed observation is $\hat {\mathbf {Y}} = \hat {\mathbf {W}} \hat {\mathbf {H}}$. Goodness of fit is defined as:

6 $$ \mathrm{Goodness~of~fit} = r(vec(Y), vec(\hat{Y})).  $$

*vec*(·) is the vectorization operation.

RMSE is also a widely used metrics calculated by the root mean squared error between the estimated and true proportions, i.e.,

7 $$ RMSE = \sqrt{\sum{(\mathbf{Y} - \hat{\mathbf{Y}})^{2}}/PN}.  $$

### Datasets

All the six datasets used in the study are publically available and can be downloaded from the Gene Expression Omnibus (GEO): Mouse-Mix data by Shen-Orr et al. [8] (accession GSE19830), Immune data by Abbas et al. [14] (accession GSE11058), EPIC (European Prospective Investigation into Cancer and Nutrition) data by Riboli et al. [49] (accession GSE51032), Aging data by Hannum et al. [50] (accession GSE40279), RA (Rheumatoid Arthritis) data by Liu et al. [51] (accession GSE42861), and Breast data [10] (accession GSE88883). The purified blood cell profiles are obtained from the R/Bioconductor package *FlowSorted.Blood.450k* and are originally obtained by Reinius et al. [26]. After the preprocessed data are downloaded from GEO, RMA are used to normalize gene expression data and quantile normalization are used to normalize the DNA methylation data.

### Implementation of the reference-free methods

We use the ged function from R package *CellMix* [69], downloaded from GitHub (https://github.com/rforge/cellmix/tree/master/pkg), for the deconf algorithm [19] implementation. We use the RefFreeCellMix function from R package *RefFreeEWAS* [20] which is obtained from its CRAN page (https://cran.r-project.org/web/packages/RefFreeEWAS/index.html). The BayesCCE Matlab toolbox is downloaded from GitHub (https://github.com/cozygene/BayesCCE). In order to incorporate BayesCCE with our algorithm, we call the matlab function from R using the *R.matlab* package [70].

When implementing BayesCCE, we set parameters *k* = 6 and *d* = 10 and select the initial variables with ReFACTor as recommended in the BayesCCE paper. In addition to high-throughput data matrix, BayesCCE accepts known patient phenotype information. We accounted for age and gender for all analyzed DNA methylation, EPIC, Aging, and RA data. We also accounted for the smoking status when analyzing the RA data. For the prior information of cell proportions used in BayesCCE, we use the true parameter values in generating simulation proportions (0.968,4.706,0.496,0.347) for four synthetic cell types, when evaluating BayesCCE in simulation study. In real data analysis with blood samples, we use the parameter (15.0727,1.8439,2.5392,1.7934,0.7240,0.7404) for granulocytes, monocytes, CD4+, CD8+, B cells, and NK cells provided by the BayesCCE paper and originally obtained from the Perioperative Medicine at UCLA’s perioperative data warehouse (PDW) [71].

## Additional files

Additional file 1 Supplementary Section S1 (Procedures to obtain cell type-specific features in simulation study); Section S2 (Simulation study for RNA-seq data); Figure S1-S17. (PDF 1526 kb)

Additional file 2 Review history. (DOCX 961 KB)

## Data Availability

The datasets analyzed in this study are available from the Gene Expression Omnibus (GEO) repository under the following accession numbers: GSE19830 [8], GSE88883 [10], GSE11058 [14], GSE51032 [49], GSE40279 [50], and GSE42861[51]. TOAST is a Bioconductor package with license GPL-2 available at https://bioconductor.org/packages/TOAST.

## Review history

The review history is available as Additional file 2.
