# Supplementary material for: TOAST: improving reference-free cell composition estimation by cross-cell type differential analysis
Source: Genome Biol. 2019 Sep 4;20:190. doi: 10.1186/s13059-019-1778-0 (PMC6727351; doi:10.1186/s13059-019-1778-0)
Supplement: Supplementary file 1 — Supplementary Section S1 (Procedures to obtain cell type-specific features in simulation study); Section S2 (Simulation study for RNA-seq data); Figure S1-S17. (PDF 1526 kb) [file 13059_2019_1778_MOESM1_ESM.pdf]

TOAST: improving reference-free cell composition estimation by cross-cell type differential analysis
Additional file 1

Ziyi Li and Hao Wu*

Department of Biostatistics and Bioinformatics, Emory University, Atlanta, GA 30322, USA.

* Correspondance: hao.wu@emory.edu.

S1. Procedures to obtain cell type-specific features in simulation study

Given the observed reference panel $\boldsymbol{W}$, which is a G by K matrix. Our goal is to obtain the top G_0 features that demonstrate cross-cell type differences. In our simulation study implementations, we specify G_0 as 1000. If more replicates are available, we can use t-test. As we only have one biological replicate for our simulation study, we use log fold change between cell types to quantify cross-cell type differences.

1. For cell type k and gene g, calculate the mean of log fold change between cell type k and all the other cell types, defined as d_{gk}. Repeat this step for gene $g = 1$ to G.
2. Sort $\boldsymbol{D_k} = (d_{1k}, \cdots, d_{Gk})$, and choose the top $[G_0/K * 1.2]$ features from the sorted $\boldsymbol{D_k}$ as $\boldsymbol{F_k}$. $[\cdot]$ is the floor operation. We choose 1.2 times the desired value because different cell types may have overlapped cross-cell type features.
3. Repeat step 1 and 2 for all cell types, and denote the obtained feature lists as $\boldsymbol{F_1}, \boldsymbol{F_2}, \cdots, \boldsymbol{F_K}$.
4. Merge $\boldsymbol{F_1}, \boldsymbol{F_2}, \cdots, \boldsymbol{F_K}$, remove replicated index and choose the top G_0 features, resulting in a combined feature list $\boldsymbol{F}$.

$\boldsymbol{F}$ is the desired cross-cell type feature list.

S2. Simulation study for RNA-seq data

We conduct simulation studies for evaluating the features selection function by TOAST for RNA-seq data. The simulation parameters are estimated from a real dataset on GEO with accession number GSE60424 (Linsley et al., 2014). This datasets has RNA-seq measurements of 6 purified blood cells, with 4 replicates for controls and for patients with an array of immune-associated diseases (type I diabetes, amyotrophic lateral sclerosis, sepsis, and multiple sclerosis patients). We only use the measurements for controls to estimate our parameters.

The detail simulation steps are listed below:

1. For cell type k and gene g, estimate mean μ_g^k and dispersion ϕ_g^k using the `estParam` function in Bioconductor package *PROPER* (Wu et al., 2014).

2. For person i, simulate the individualized underlying cell type specific expressions m_{gi}^k from a Gamma distribution.
$$m_{gi}^k \sim \Gamma(\mu_g^k, \textbf{scale} = \frac{\phi_g^k}{1-\phi_g^k})$$

3. Simulate the mixing proportions from a Dirichlet distribution with parameters $(0.89, 4.11, 0.47, 0.33, 0.61)$.

4. For person i, mix the cell type specific expressions to generate the expression for mixed samples.

5. Generate read count Y_{gi} from Poisson distribution.
$$Y_{gi}|m_{gi} \sim Poisson(m_{gi})$$

After observations $Y = (Y_{gi})$ are generated, we apply the proposed method on the raw counts, and the results are presented in Figure S16. All results are summarized over 20 Monte Carlo datasets.

Reference to Section S2

Linsley, P. S., Speake, C., Whalen, E., & Chaussabel, D. (2014). Copy number loss of the interferon gene cluster in melanomas is linked to reduced T cell infiltrate and poor patient prognosis. PloS one, 9(10), e109760.

Wu, Hao, Chi Wang, and Zhijin Wu. "PROPER: comprehensive power evaluation for differential expression using RNA-seq." Bioinformatics 31.2 (2014): 233-241.

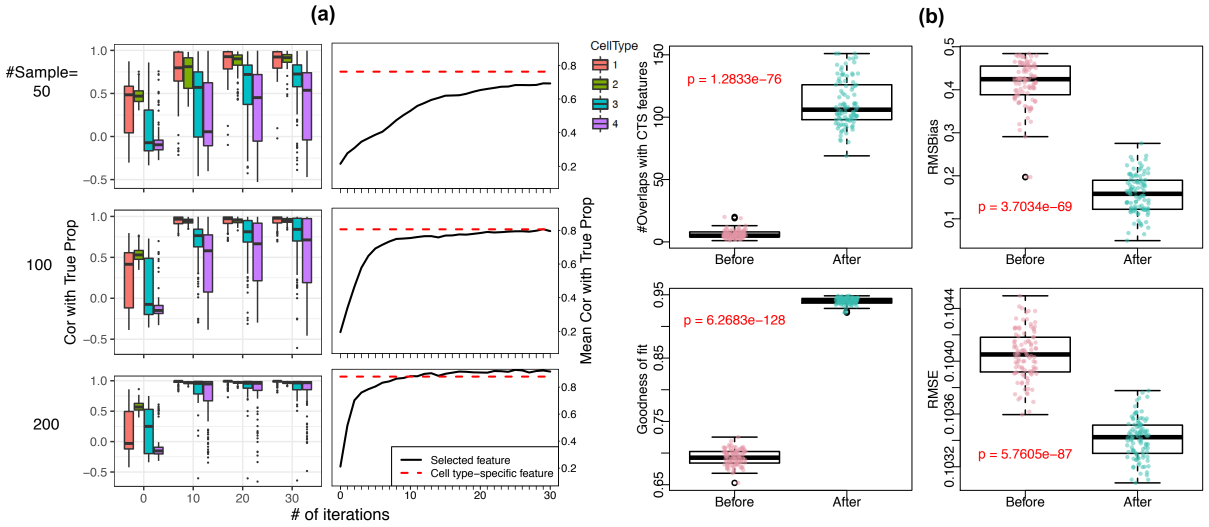

Figure S1: Results of the simulation study based on DNA methylation 450K dataset (GSE35069). Four cell types are used here to generate simulation data by combining CD8T, CD4T and NK to one cell type. Figure (a) shows the correlations between estimated proportions versus true proportions by number of iterations. Left panel of (a): boxplot of correlations for four cell types by number of iterations. Right panel of (a): mean correlations across four cell types by number of iterations. Figure (b) shows the number of overlaps with cell type-specific (CTS) markers before and after iterations in the top left panel, the root mean squared bias (RMSBias) in the top right panel, the goodness of fit in the bottom left panel, and the root mean squared error (RMSE) in the bottom right panel. P values in each panel are obtained using paird t-test. Red font indicates being statistical significant. Top 1001-2000 most variable features are selected as initial features. Baseline performance is presented in the "number of iterations = 0" columns in (a) and "Before" columns in (b).

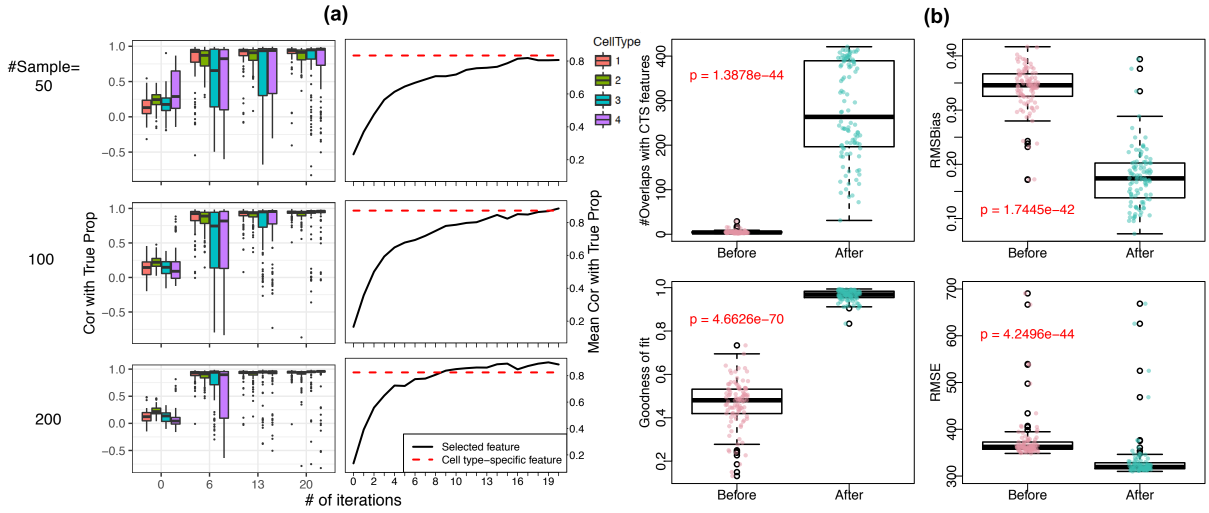

Figure S2: Results of the simulation study based on gene expression microarray dataset (GSE19830). Figure (a) shows the correlations between estimated proportions versus true proportions by number of iterations. Left panel of (a): boxplot of correlations for four cell types by number of iterations. Right panel of (a): mean correlations across four cell types by number of iterations. Figure (b) shows the number of overlaps with cell type-specific (CTS) markers before and after iterations in the top left panel, the root mean squared bias (RMSBias) in the top right panel, the goodness of fit in the bottom left panel, and the root mean squared error (RMSE) in the bottom right panel. P values in each panel are obtained using paird t−test. Red font indicates being statistical significant. Top 1000 most variable features are selected as initial features. Baseline performance is presented in the "number of iterations = 0" columns in (a) and "Before" columns in (b).

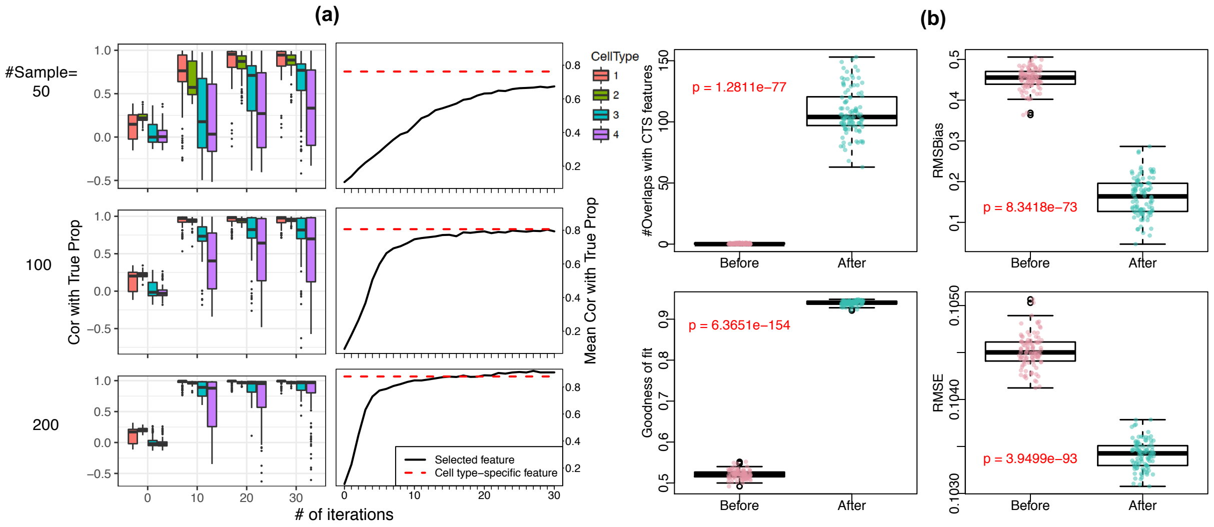

Figure S3: Results of the simulation study based on DNA methylation 450K dataset (GSE35069). Four cell types are used here to generate simulation data by combining CD8T, CD4T and NK to one cell type. Figure (a) shows the correlations between estimated proportions versus true proportions by number of iterations. Left panel of (a): boxplot of correlations for four cell types by number of iterations. Right panel of (a): mean correlations across four cell types by number of iterations. Figure (b) shows the number of overlaps with cell type-specific (CTS) markers before and after iterations in the top left panel, the root mean squared bias (RMSBias) in the top right panel, the goodness of fit in the bottom left panel, and the root mean squared error (RMSE) in the bottom right panel. P values in each panel are obtained using paird $t-$test. Red font indicates being statistical significant. Top 1000 most variable features are selected as initial features. Baseline performance is presented in the "number of iterations = 0" columns in (a) and "Before" columns in (b).

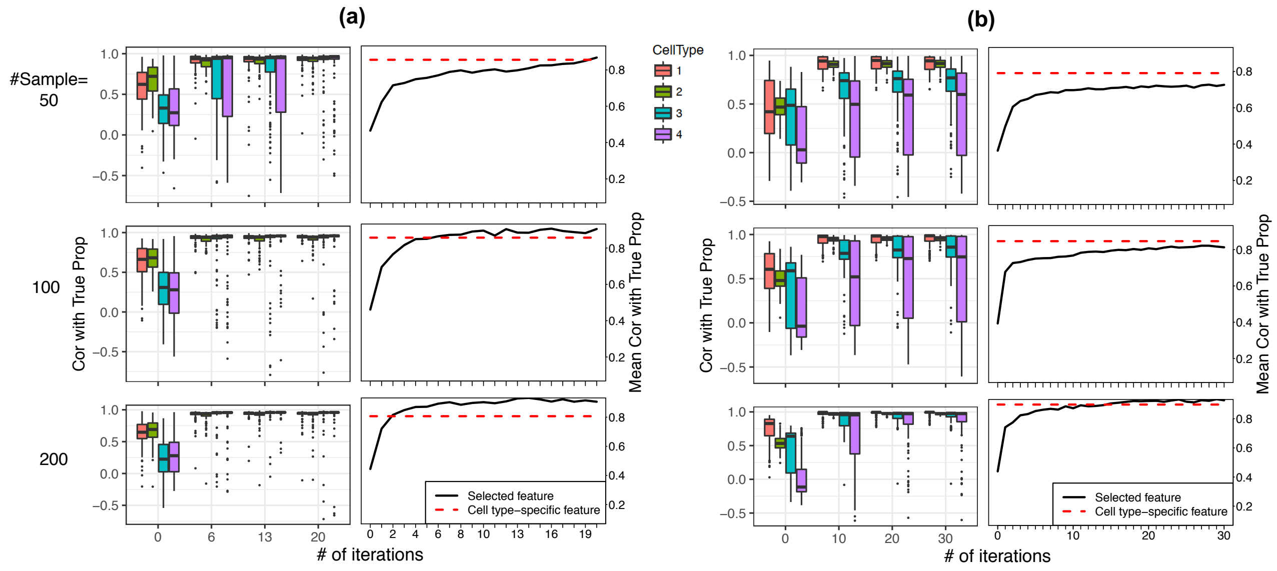

Figure S4: The correlations between estimated proportions versus true proportions by number of iterations. Results are based on simulation studies with gene expression microarray dataset (GSE19830) (Figure a) and DNA methylation 450K dataset (GSE35069) (Figure b). 1000 randomly selected features are used as initial features. Baseline performance is presented in the "number of iterations = 0" columns in (a) and (b).

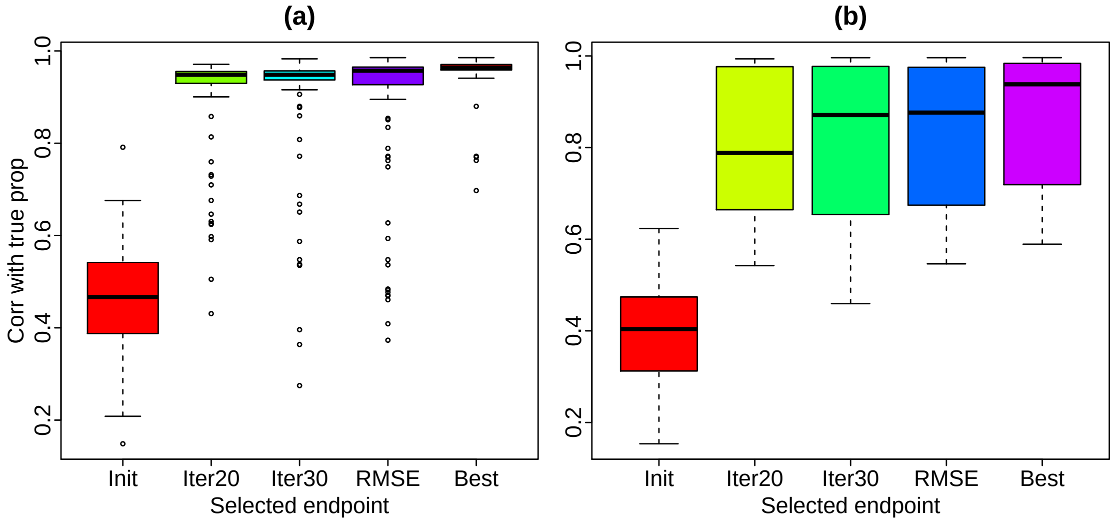

Figure S5: Smallest root mean squared error (RMSE) serves as a a good endpoint selection approach for the proposed method based on simulation study when randomly selected features are used as initial features. Figure (a) is the results based on gene expression simulation studies and Figure (b) is based on DNA methylation simulation studies. All plots are summarized over 100 Monte Carlo experiments. The "Init" columns are performance of reference-free deconvolution without applying TOAST (baseline performance).

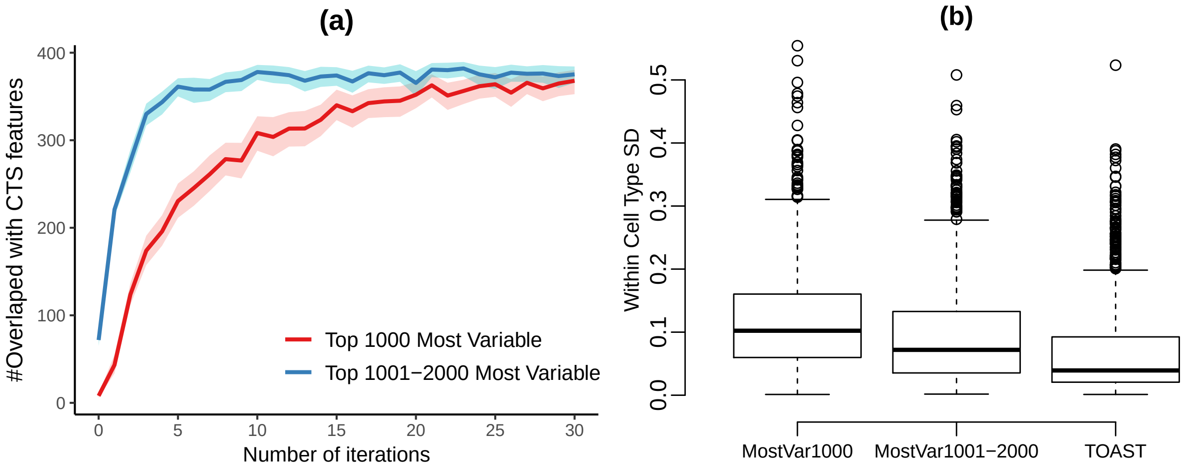

Figure S6: Exploration of initial marker selections. Figure (a) plot number of selected features overlapped with true cell type-specific (CTS) features by number of iterations. The "number of iterations = 0" column is the baseline performance without using TOAST. Figure (b) is the boxplot of within-cell type standard deviations by different selections of features. MostVar1000 is the top 1000 most variable features. MostVar1001-2000 is the second top 1000 most variable features. TOAST is the 1000 features from the proposed method using smallest RMSE as iteration end point. Figure (a) is based on 100 simulation datasets with sample size 100 in the gene expression simulation study. Figure (b) is obtained based on the real gene expression dataset *Mouse-Mix* downloaded from GEO with accession number GSE19830. Presented results in (b) are based on liver tissue.

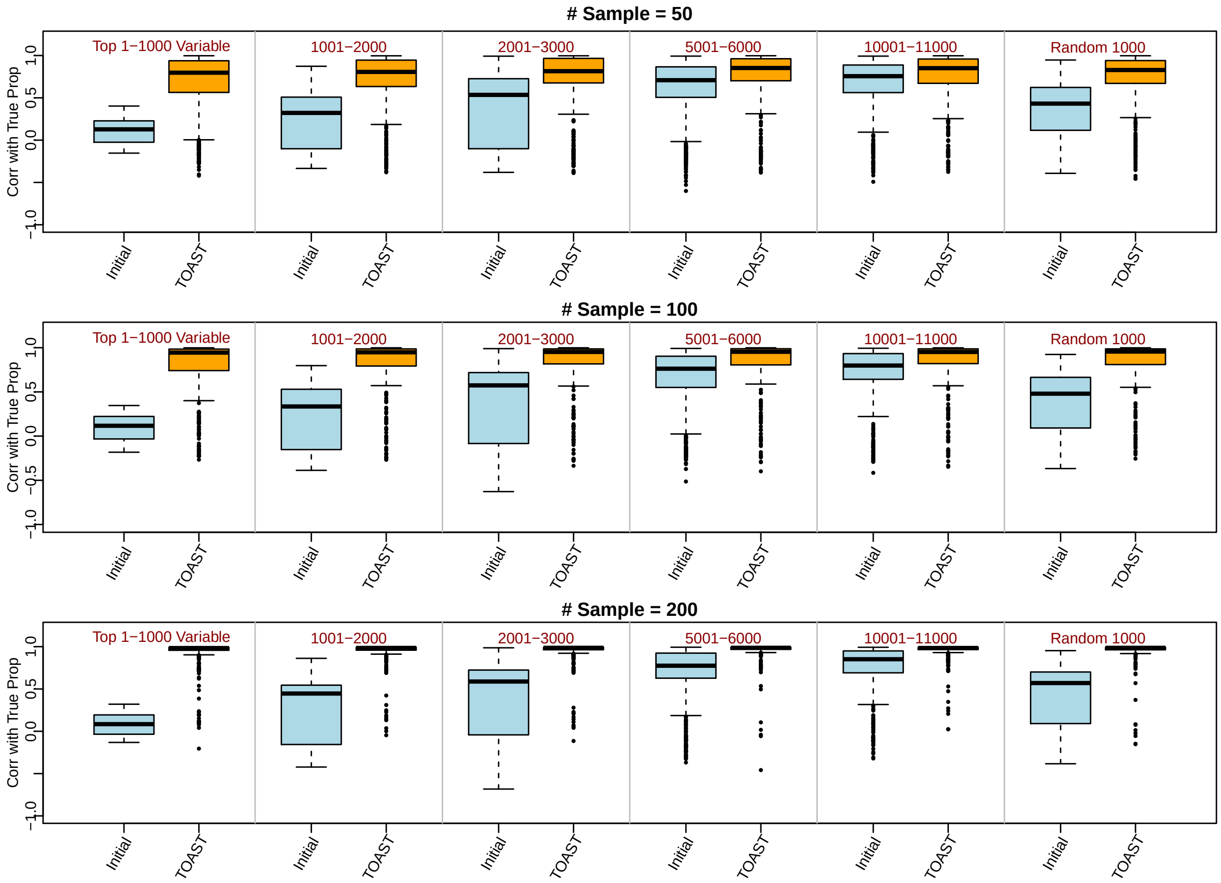

Figure S7: TOAST is stable with different initial features selections in DNA methylation simulation studies. The panels from top to bottom correspond to sample sizes 50, 100 and 200. The panels from left to right correspond to different methods of selecting initial features: Top 1-1000 variables is to select the top 1000 most variable features, 1001-2000 is to select the top 1001-2000 most variable features, similarly 2001-3000, 5001-6000, and 10001-11000 are to select the top 2001-3000, 5001-6000, and 10001-11000 most variable features. Random 1000 is to randomly select 1000 features as initial features. In each panel, "Initial" and "TOAST" correspond to reference-free deconvolution results without and with TOAST. The presented results are summarized over 100 Monte Carlo experiments.

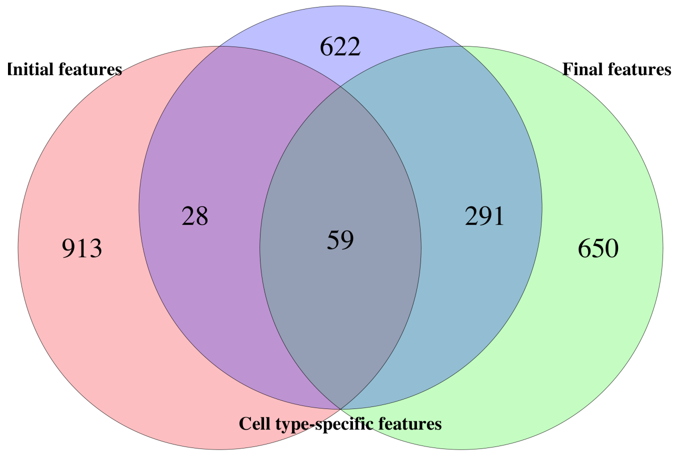

Figure S8: Venn diagram of initial features, cell type-specific features and final features based on gene expression simulation data.

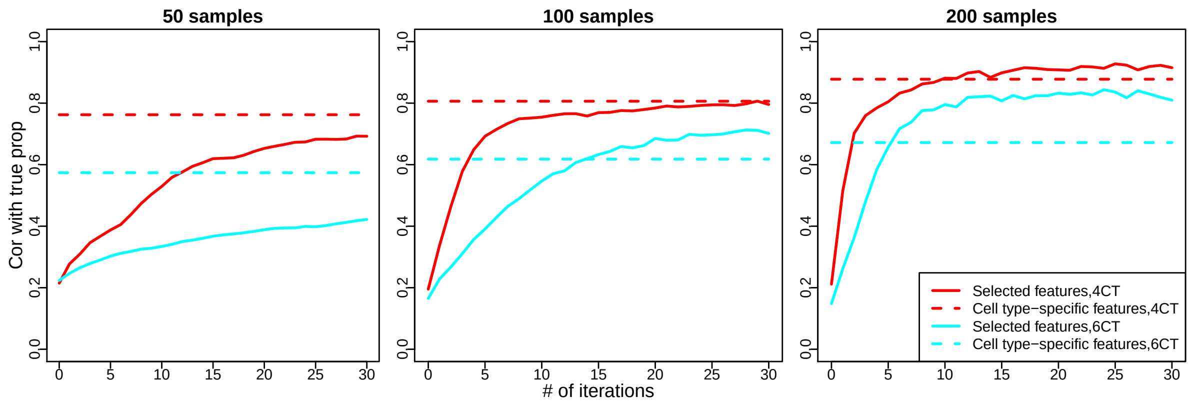

Figure S9: Correlations of estimated proportions with true proportions versus number of iterations. Results are based on DNA methylation 450K dataset (GSE35069) and correlations are average across all cell types. Red lines represent results from simulation with four cell types (CD8T+CD4T+NK, Bcell, Gran, and Mono). Blue lines represent results from simulation with six cell types (CD8T, CD4T, NK, Bcell, Gran, and Mono). Cell type-specific markers are chosen from pure tissue reference matrix. Top 1001-2000 most variant CpGs are used as initial features. The "number of iterations = 0" columns are baseline performance without applying TOAST.

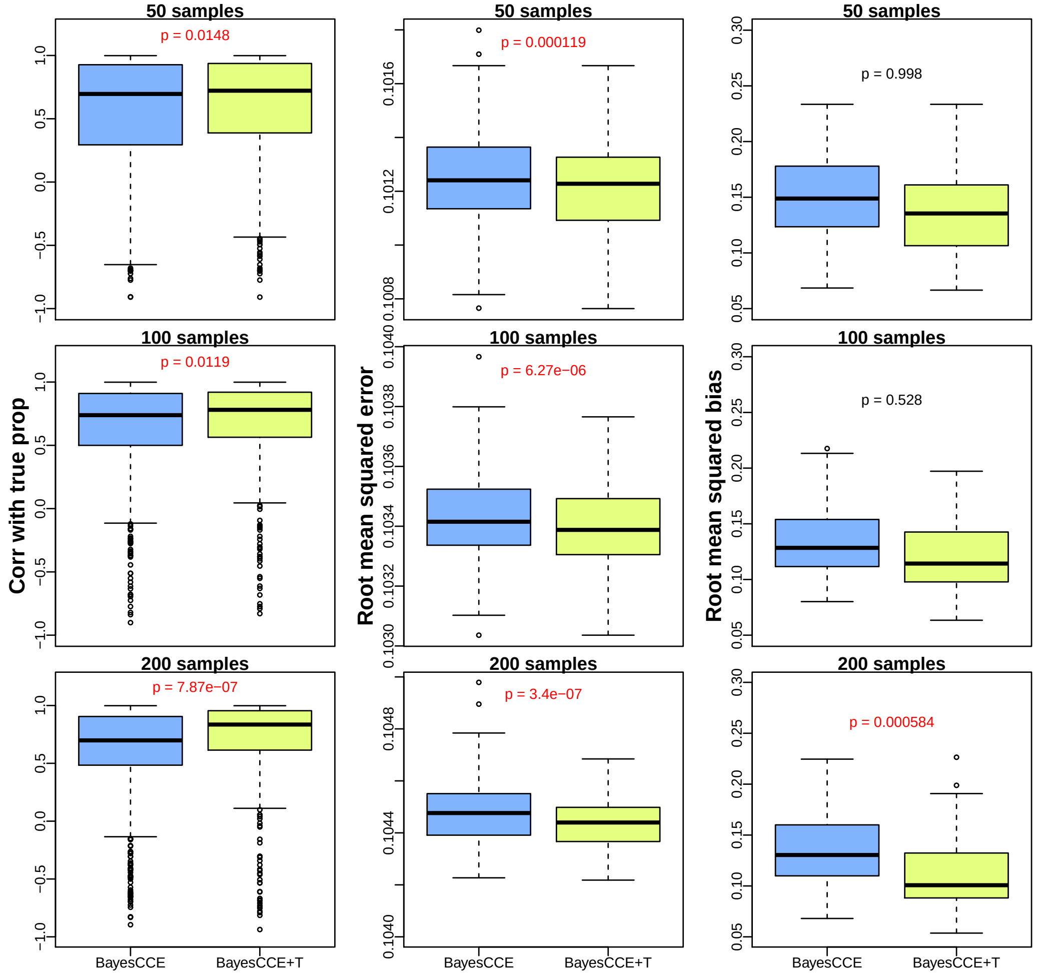

Figure S10: The proposed method could improve the deconvolution performance of BayessCCE in simulation studies. BayesCCE is the performance of BayesCCE without applying TOAST (baseline performance). BayesCCE+T is the BayessCCE algorithm incorporated with the proposed method TOAST. Results are based on 100 DNA methylation simulation datasets with four cell types. Panels from left to right demonstrate correlations of estimated proportions versus true proportions (Corr with true prop), root mean squared error, and root means squared bias. Panels from top to bottom correspond to sample size 50, 100, and 200. The demonstrated p values are obtained from paired t−test. Red fonts indicate significant test results. Boxplots are summarized from 100 Monte Carlo experiments.

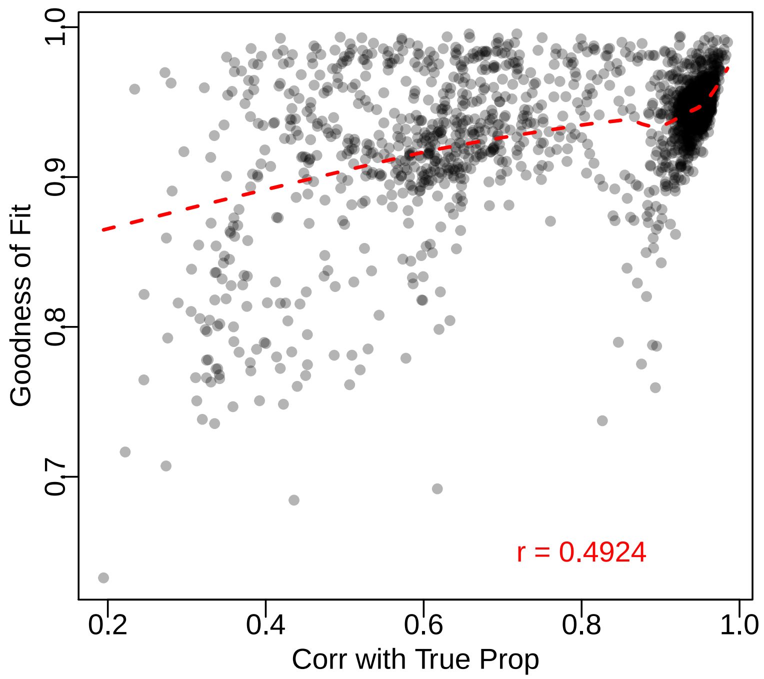

Figure S11: Scatterplot of goodness of fit versus correlations with true proportions in gene expression simulation studies. The red dotted curve is the best fit curve by loess. "r" is the Pearson's correlation coefficient.

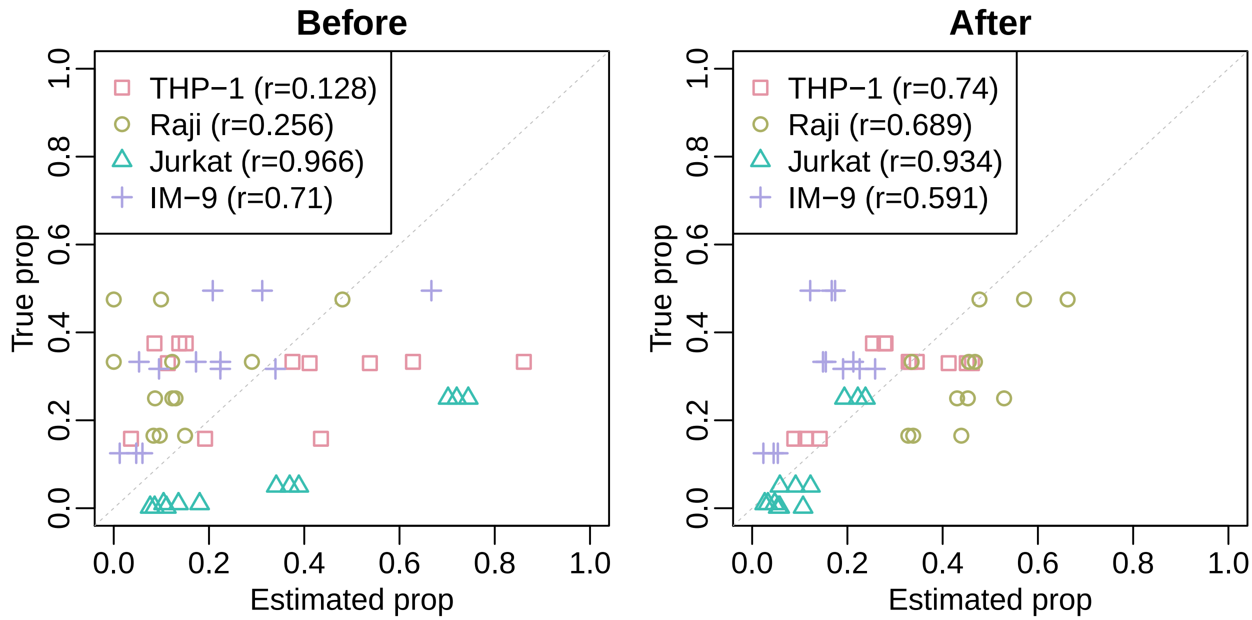

Figure S12: Proportion estimations from the Immune dataset (GSE11058). Left panel: estimated proportions versus true proportions (baseline performance). Right panel: estimated versus true proportions after updating feature selection by the proposed algorithm.

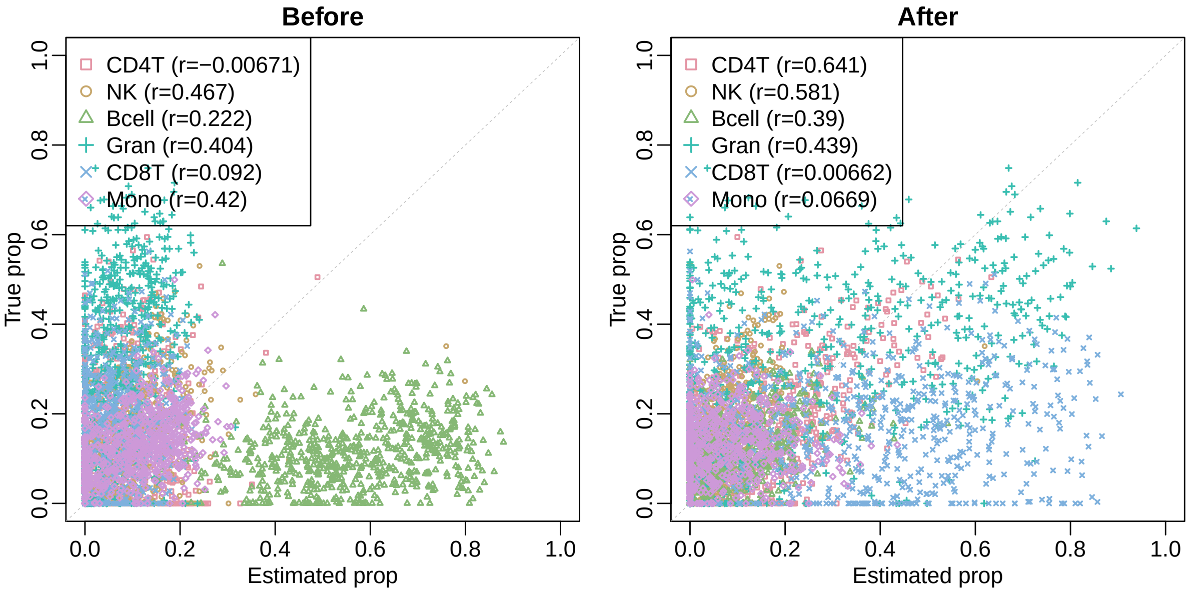

Figure S13: Proportion estimations from the Aging dataset (GSE40279). Left panel: estimated proportions versus true proportions (baseline performance). Right panel: estimated versus true proportions after updating feature selection by the proposed algorithm. "True" proportion of the Aging dataset is obtained using RB deconvolution method *EpiDISH*.

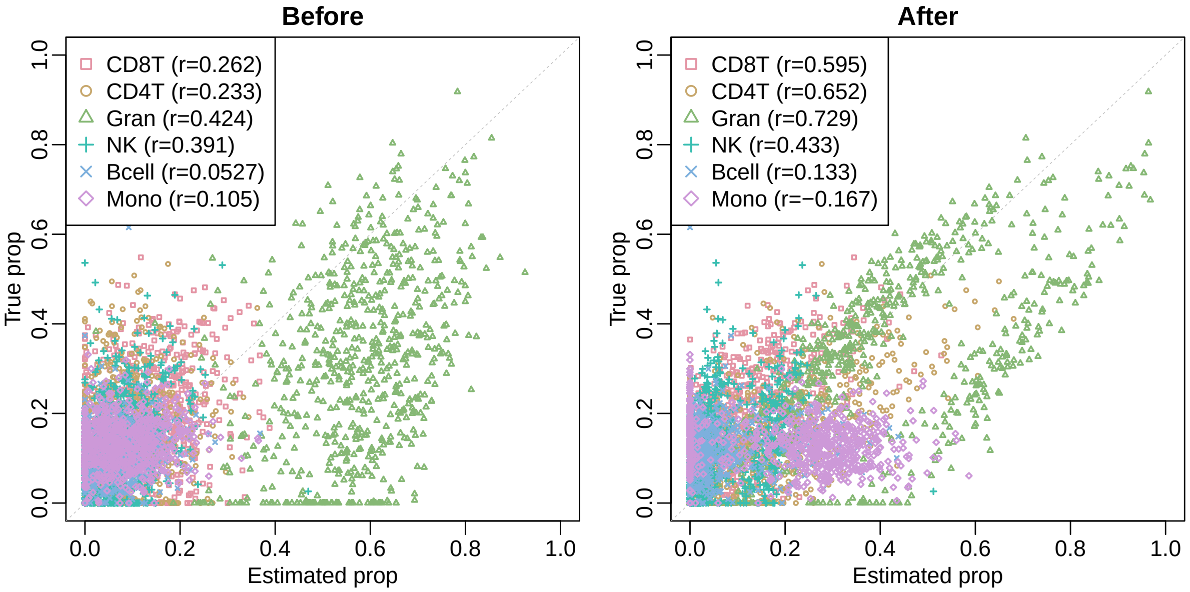

Figure S14: Proportion estimations from the RA dataset (GSE42861). Left panel: estimated proportions versus true proportions (baseline performance). Right panel: estimated versus true proportions after updating feature selection by the proposed algorithm. "True" proportion of the RA dataset is obtained using RB deconvolution method *EpiDISH*.

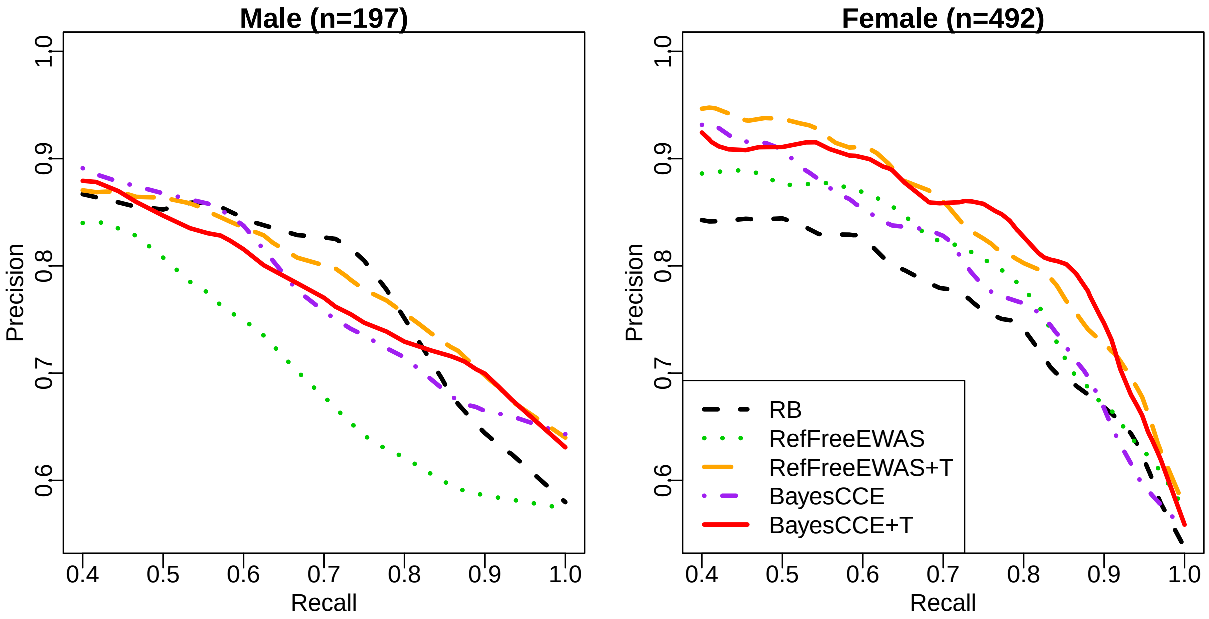

Figure S15: Precision recall curve from the analysis of RA dataset by gender. There are a total 197 males and 492 females. RB, RefFreeEWAS, RefFreeEWAS with the proposed method TOAST (RefFreeEWAS+T), BayesCCE and BayesCCE with TOAST (BayesCCE+T) are used to estimate mixing proportions. 10-fold cross validation is used to generate prediction results.

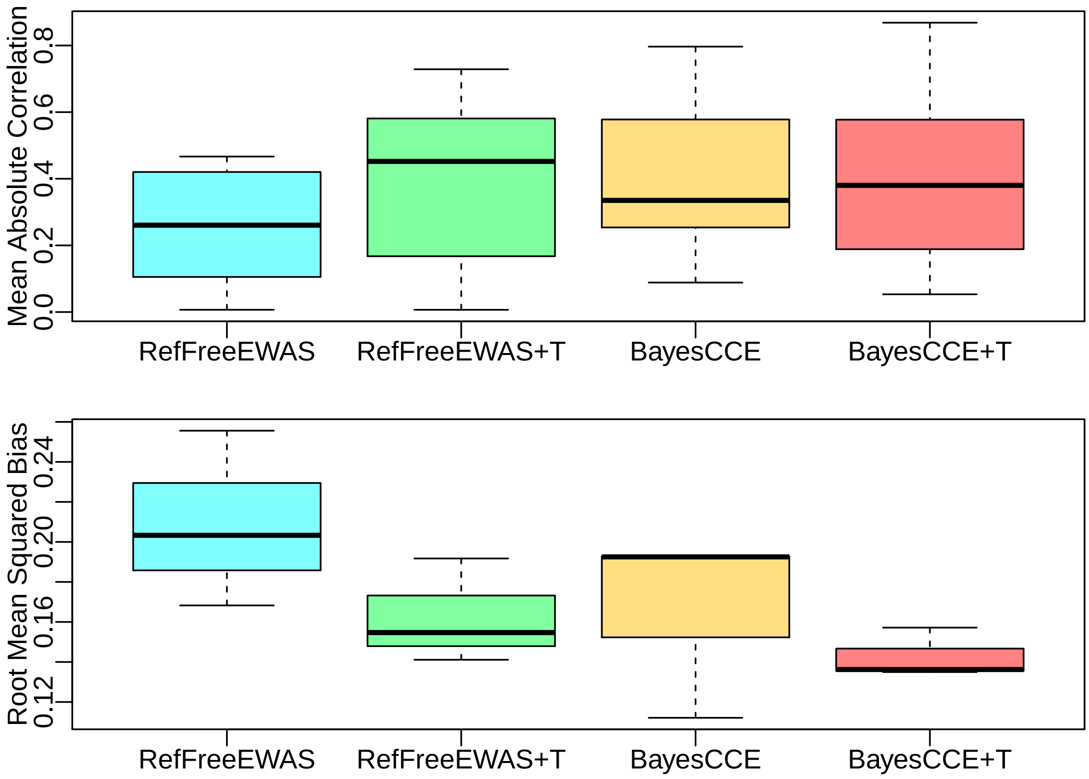

Figure S16: The proposed method improves the performance of RefFreeEWAS and BayesCCE in deconvolving real DNA methylation datasets (EPIC, Aging, and RA studies). RefFreeEWAS, RefFreeEWAS with the proposed method TOAST (RefFreeEWAS+T), BayesCCE and BayesCCE with TOAST (BayesCCE+T) are used to estimate mixing proportions. Top and bottom panels show the mean absolute correlations and root mean squared bias with reference-based estimated proportions.

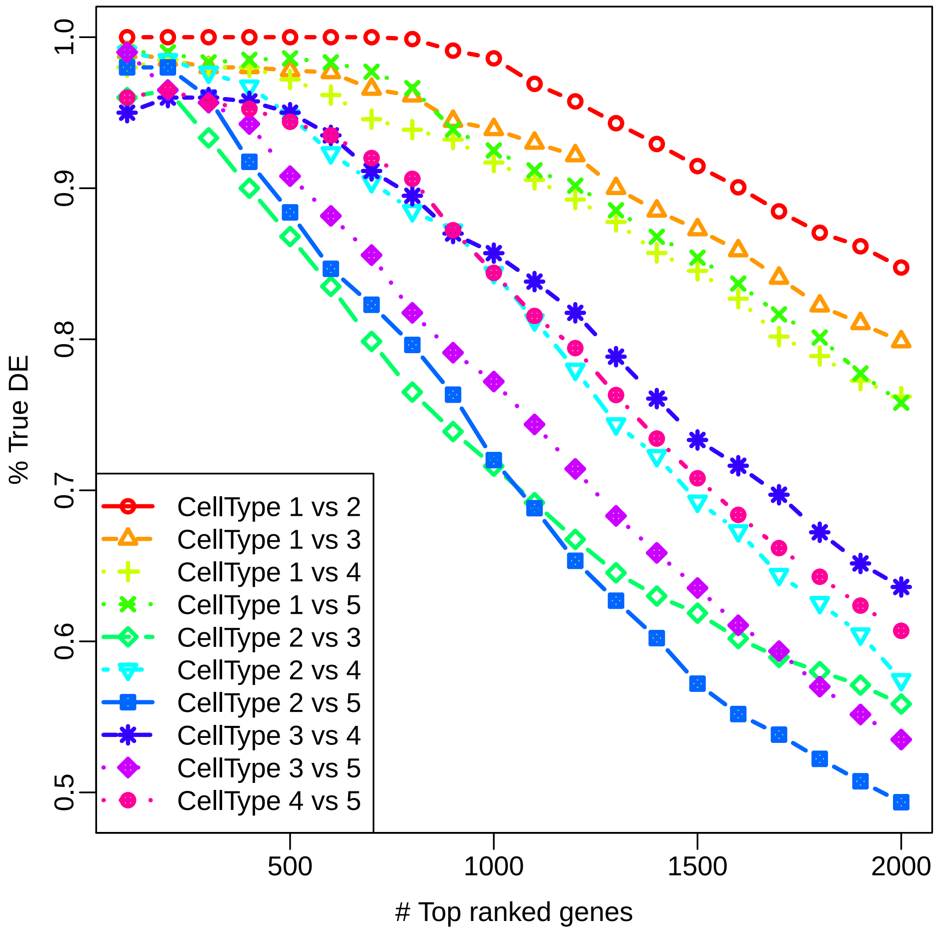

Figure S17: Accuracy of detecting cross-cell type differential signals by TOAST using RNA-seq simulation dataset. Results are summarized over 20 RNA-seq simulated dataset.
