# Supplementary material for: TOAST: improving reference-free cell composition estimation by cross-cell type differential analysis
Source: Genome Biol. 2019 Sep 4;20:190. doi: 10.1186/s13059-019-1778-0 (PMC6727351; doi:10.1186/s13059-019-1778-0)
Supplement: Supplementary file 2 — Review history. (DOCX 961 KB) [file 13059_2019_1778_MOESM2_ESM.docx]

Review History

**First round of review**

**Comments to author and authors’ point-by-point responses to the reviewers’ comments:**

**Reviewer 1**

Li and Wu described an improved method for reference-free (RF) deconvolution implemented in TOAST (TOols for the Analysis of heterogeneouS Tissues). Based on a previously published statistical framework, the authors focused on the proportion estimation step and aimed to improve feature selection of RF methods. They proposed a method that iterates RF deconvolution and TOAST-based cross-cell type differential analysis. Post iterations, cell-type-specific features would be refined to better estimate cell proportions for cross-sample cell-type-specific differential analysis. Results from both simulations and real datasets suggest that they obtained better cell type proportion estimations for microarray-based bulk profiles.

The method seems solid and can be useful if more thoroughly tested and adapted for common practices. The improvement seems incremental and the manuscript lacks rigor and clarification. A few specific comments:

(Comment 1) What does devolution look like without using TOAST? There isn't a baseline to which the addition of feature selection can be compared (it might be before and after for figure 2b, but I'm not sure).

*Response: Thank you for the question. The deconvolution performance is demonstrated in the “# of iterations = 0” columns in Figure 1(a), 2(a), S1(a), S2(a), S3(a), the “Before” columns in Figure 1(b), 6, 7(a), 7(b), S1(b), S2(b), S3(b), S6, S7, S8, the “Selected endpoint = Init” columns in Figure 4(b) and 4(d), the “BayesCCE” columns in Figure 5, 8(b), S5, S9, and the “RefFreeEWAS” columns in Figure 8(b) and S9. We have added related explanations to manuscript to clarify the baseline (Legends of Figure 2, 5, 6, 7, 8, S1, S2, S4, S4, S5, S6, S9, S10, S11, S12, S13).*

How are the real cell type-specific features obtained? Why is it that TOAST can perform better than the true cell type-specific features?

*Response: In simulation studies, the cell type-specific features are obtained from the reference panel, i.e. pure tissue profile matrix. For each feature, we compare the expression level in reference panel from one cell type against the mean of the other cell types and then calculate the absolute log fold change (because the reference panel does not have biological replicates). The top features with largest absolute log fold change are chosen for each cell type, and together they constitute the cell type-specific feature list. We have included a section in Supplementary materials to explain the detailed steps to obtain cell type-specific features from simulation datasets (Supplementary materials, Section S1).*

*TOAST can achieve comparable or even better performance than cell type-specific features because of the biological variances and biases of the reference panel (pure cell type profiles). In real world scenario, reference panels (W) are often obtained from a population different from the samples being studied. Thus, there could be biases in the reference. TOAST obtains cell type-specific features based on the mixed measurements from the current samples. When the biases in the existing reference panels are large, TOAST can achieve better performance than using external reference panel. We have added some discussion on this point at page 4 paragraph 1.*

What does sample size exactly mean? My impression is that it means number of complex samples of the same type being sequenced. In that case, do sample sizes of 50+ make sense to test? What are sample sizes that studies usually have?

*Response: Your understanding is correct. Sample size in our study refers to the number of heterogeneous samples measured and used for deconvolution. The first sentence in our manuscript indicates that the sample deconvolution problem is important for “large-scale clinical studies … to profile biological samples collected from human subjects”. In such studies, sample sizes of 50+ is not only reasonable, but also necessary. In our opinion, the small sample size study (e.g., <5 replicates in each biological condition) is only reasonable for very homogeneous samples, such as cell lines or model organisms. To study heterogeneous and complex diseases in human subjects, large sample size is necessary to provide enough power to identify disease biomarkers and therapeutic targets. This is the reason why most serious studies of human diseases has large sample sizes, for example, TCGA for cancers, ROSMAP for neurodegenerative diseases, etc.*

*For datasets used in this manuscript, the Breast dataset has 100 subjects, the EPIC dataset has 424 cancer-free subjects, the Aging dataset has 656 subjects, and the RA dataset has 689 subjects. For another example, in the BayesCCE (a state-of-art reference-free deconvolution method) paper by Rahmani et al. Genome Biology (2018), the four real datasets (Hannum et al., Liu et al., Hannon et al. I, Hannon et al. II) have sample sizes 650, 658, 638 and 665.*

*We have added discussions about sample size in the Discussion section (page 10 paragraph 1).*

*References for Comment 1:*

*Rahmani, Elior, et al. "BayesCCE: a Bayesian framework for estimating cell-type composition from DNA methylation without the need for methylation reference." Genome biology 19.1 (2018): 141.*

*Hannum, Gregory, et al. "Genome-wide methylation profiles reveal quantitative views of human aging rates." Molecular cell49.2 (2013): 359-367.*

*Liu, Yun, et al. "Epigenome-wide association data implicate DNA methylation as an intermediary of genetic risk in rheumatoid arthritis." Nature biotechnology 31.2 (2013): 142.*

*Hannon, Eilis, et al. "An integrated genetic-epigenetic analysis of schizophrenia: evidence for co-localization of genetic associations and differential DNA methylation." Genome biology 17.1 (2016): 176.*

(Comment 2) Why would the second 1000 most variable features overlap with more cell type-specific features than the first 1000 most variable features? There doesn't seem to be any reason to use variability of a feature as a metric to selecting initial features as they don't overlap with cell type-specific features. It would be helpful to know the total number of cell type-specific features so that the number of overlaps with those features is more meaningful.

*Response: Thank you for the comment. First of all, the variability of features has been commonly used as an indicator of how “informative” a feature is for sample mixing (Teschendorff and Relton, Nature Reviews Genetics, 2017). A survey of existing publications with reference-free deconvolution applications shows largest variability is commonly (8 out of 10) used in reference-free deconvolution analysis (Table 1). Indeed it is interesting to observe that the second 1000 most variable features overlap with more cell type-specific features than the first 1000 most variable features. As illustrated in Figure 3(b), we believe a large portion of variations of the most variable features are from within cell type variations, which is different from the characteristics of cell type-specific features (i.e. large cross cell type variations).*

*Furthermore, we want to emphasize that the initial feature selection is not a focal point of our work. We have more detailed responses and results regarding to this point for comment 4 below, where we show that TOAST improves the RF estimation regardless of the initial markers selected.*

*We identify a total of 1000 cell type-specific features from the reference panel. We have added the information to manuscript to clarify this (page 4 paragraph 1).*

*Reference to Comment 2:*

*Teschendorff, Andrew E., and Caroline L. Relton. "Statistical and integrative system-level analysis of DNA methylation data." Nature Reviews Genetics 19.3 (2018): 129.*

(Comment 3) For figure 4a and c, would including the correlation r make sense? It just seems like there's a lot of points so that the p-value is low, which makes it rather meaningless to me. The best fit line (?) doesn't even represent the majority of points in 4a.

*Thanks for the comment. We also agree that the p-value in this plot does not convey much. We have taken your suggestion and replace the p-value by correlation r in Figure 4(a) and (c).*

(Comment 4) The init performs poorly in comparison to their method, but it would seem to me that there's no reason to believe it would perform well since the starting feature (top 1001-2000 most variable) is an improper starting point based on the previous figure. Overall, it is a somewhat odd choice to use the most variable genes/regions as initial features (top 1000 or 1001 to 2000). This selection lacks justification when initially introduced, and the author also noted later that those features are the ones that show mainly within cell type variation. Although it is mentioned in the discussion that the choice of initial features is beyond the scope of this study, some justification/explanation should be made during the introduction. Or using all or some random regions/genes with high signal as initial features might be a more neutral and less confusing choice.

Since the initial feature selection lacks a strong justification, changes in metrics using before and after algorithm selected features cannot sufficiently demonstrate the performance of the algorithm.

*Response* *First, we want to argue that using the most variable features is not an “odd” choice. In fact, it is the most natural data-driven choice when there’s no prior biological knowledge. Intuitively, cell type specific features have distinct profiles among different cell types, thus the mixed signal will be dependent of the mixing proportions. When the mixing proportions vary among samples, the mixed signals will exhibit large variation. On the other hand, features that are not cell type specific have roughly the same profiles in different cell types, thus the mixed signals will be independent of the mixing proportions and have small variation among samples. This is the rationale for using the most variable features in deconvolution from most methods.*

*As mentioned in the response to comment 2, selecting feature by variability is a very popular unsupervised feature selection strategy in (epi)genomics studies (Teschendorff and Relton, Nature Reviews Genetics, 2017; Van’t Veer et al., Nature, 2002; Wang et al., BMC bioinformatics, 2014; Zhuang et al., BMC bioinformatics, 2012). Using the most variable features in reference-free deconvolution is officially recommended in a number of existing reference-free deconvolution publications (Rahmani et al. Nature methods, 2016; Rahmani et al. Genome Biology, 2018; Houseman et al. BMC bioinformatics 2016). Moreover, a survey of existing applications of reference-free deconvolution reveals that selecting the top variable features is the most common (8 out of 10) approach to conduct feature selection (Table 1). We have added related explanations to the introduction section of manuscript (page 2 paragraph 2).*

*That being said, we appreciate the reviewer’s suggestion about using random features as initial feature to demonstrate robustness. We have re-run the simulation studies for gene expression and DNA methylation data by choosing 1000 random features as initial markers. Figure S5 (also shown below) is the corresponding plots to Figure 4(b) and (d) with randomly selected initial markers for evaluating the endpoint selections. Again, we observe substantial improvements from with (Iter20, Iter30, RMSE, Best) and without (Init) applying TOAST.*

*Figure S4 is the plot for gene expression and DNA methylation simulation studies using 1000 randomly selected initial features. Comparing without (# of Iterations = 0) to with (# of Iterations >0) applying TOAST, we observe substantial improvements for correlations with true proportions, and across most of the metrics. We have added some explanations in the manuscript (page 5 paragraph 1)*

*Gene expression and DNA methylation simulation studies with 1000 randomly selected features:*

*In addition, we have explored the impact of different initial feature selection on TOAST in both gene expression and DNA methylation data. We considered the top most variable 1000, 1001-2000, 2001-3000, 5001-6000, 10001-11000 features as well as 1000 randomly selected features. All results are summarized over 100 Monte Carlo datasets. As demonstrated in Figure 3 (previous Figure 3 has been moved to Figure S6) and S7, no matter which method is used to select initial markers, applying TOAST (all “TOAST” columns) demonstrates substantial improvements over without applying TOAST (all “Initial” columns). In addition, the results after applying TOAST are stable across different sample sizes (rows), different initial feature selections (columns), and data types (Figure 3 and S7).*

*All the above results demonstrate the robustness and favorable performance of TOAST. We have added additional descriptions and discussions in the “Initial feature selection” section (page 4-5) and Discussion section (page 10 paragraph 3).*

*Gene expression results:*

*DNA methylation results:*

*Reference to Comment 4:*

*Teschendorff, Andrew E., and Caroline L. Relton. "Statistical and integrative system-level analysis of DNA methylation data." Nature Reviews Genetics 19.3 (2018): 129.*

*Van't Veer, Laura J., et al. "Gene expression profiling predicts clinical outcome of breast cancer." nature 415.6871 (2002): 530.*

*Wang, Xinhui, et al. "Non-specific filtering of beta-distributed data." BMC bioinformatics 15.1 (2014): 199.*

*Zhuang, Joanna, Martin Widschwendter, and Andrew E. Teschendorff. "A comparison of feature selection and classification methods in DNA methylation studies using the Illumina Infinium platform." BMC bioinformatics 13.1 (2012): 59.*

*Rahmani, Elior, et al. "Sparse PCA corrects for cell type heterogeneity in epigenome-wide association studies." Nature methods 13.5 (2016): 443.*

*Rahmani, Elior, et al. "BayesCCE: a Bayesian framework for estimating cell-type composition from DNA methylation without the need for methylation reference." Genome biology 19.1 (2018): 141.*

*Houseman, E. Andres, et al. "Reference-free deconvolution of DNA methylation data and mediation by cell composition effects." BMC bioinformatics 17.1 (2016): 259.*

(Comment 5) The real data section doesn't explain how sample size affects the results. This should be important not every project is going have/want to do 50+ samples. The sample size used in the simulation is way bigger than the number of samples in common real studies. As the authors noted that the sample size influences the iteration required for the algorithm to converge, a simulation with less than 5 samples (ideally a two to three samples) should be conducted.

*Response* *Thanks for sharing your concern. First, we agree that not every project has more than 50 samples. However, we echo our response to Comment 1 (part 3) that many studies have hundreds of samples and emphasize that our method is designed for large-scale clinical studies. We believe reference-free deconvolution methods are generally designed and suitable for large studies. As evidence of this argument, most (if not all) reference-free deconvolution methods are tested on large-scale datasets (Sample sizes in BayesCCE paper (2018): 650, 658, 638, 665; ReFACTor (2016): 489, 689; RefFreeEWAS (2016): 725, 402, 297, 203, 119, etc.).*

*Second, our real data sections show applications of the proposed method on data with relatively small sample size (Mouse-mix: 33, Immune: 11). These two datasets have small sample sizes due to the high cost of cell sorting techniques, and at the same time applying cell sorting technology provides ground truth for proportions. Figure 7(a) and S12 shows improvements after applying TOAST. And we observe greater improvement for Mouse-mix data than Immune data (the former has larger sample size).*

*Furthermore, for data set with even smaller sample size (for example, less than 5 in each group), reference-free method is not recommended and one has to rely on reference-based method or partial reference-free deconvolution methods for proportion estimation, which utilize external information such as reference panel, marker genes, and known proportion priors. In fact, we think to study complex and heterogeneous sample by such small sample size is not advisable, for it won’t have enough power to overcome the heterogeneity and detect signals.*

*We have added discussions about sample size in the Discussion section of manuscript (page 10 paragraph 1).*

*References for Comment 5:*

*Rahmani, Elior, et al. "BayesCCE: a Bayesian framework for estimating cell-type composition from DNA methylation without the need for methylation reference." Genome biology 19.1 (2018): 141.*

*Rahmani, Elior, et al. "Sparse PCA corrects for cell type heterogeneity in epigenome-wide association studies." Nature methods 13.5 (2016): 443.*

*Houseman, E. Andres, et al. "Reference-free deconvolution of DNA methylation data and mediation by cell composition effects." BMC bioinformatics 17.1 (2016): 259.*

(Comment 6) Is the difference in RB and RF estimates for RA data simply due to sex-specific differences? It would helpful to know if the same trend is observed when only considering male patients and controls.

*Response* *This is a great comment. As suggested, we have summarized our results for males and females separately in Figure S15 (also shown below).*

*First, in both male and female groups, we observe comparable or improvements after applying TOAST (yellow and red lines) over without TOAST (green and purple lines). These results again demonstrate the favorable and robust performance of the proposed method.*

*On another note, we observe greater improvements by using TOAST in females than in males. In addition, we observe different trends in comparing reference-based versus reference-free results. In male populations, there is minimum advantage in reference-free deconvolution methods (colored lines) over reference-based method (black line), while the advantage is more profound in female group and overall population (Figure 8 and S15). We believe this could be explained through two aspects:*

*1. Gender differences in RA disease etiology. It has been reported in existing publications that females have higher rates of RA occurrences compared to males (sex ratio is around 3:1), and female patients who suffered from RA disease have more severe symptoms compared to male patients (Affleck et al., PAIN, 1999; Kvien, Pharmacoeconomics, 2004; Ahlmén et al., Annals of the rheumatic diseases, 2010). The differences in disease etiology could lead to different levels of proportion alterations among males and females. Thus, it is possible that predicting disease status using proportions is harder in male than females.*

*2. Differences in sample size. The current RA data have 197 males (96 controls and 101 patients) and 492 females (239 controls and 253 patients). We suspect that the larger sample size in female lead to more accurate proportion estimation in female samples, which subsequently results in better prediction results.*

*We have included related descriptions and discussions in Result section (page 9 paragraph 3).*

*References to Comment 6:*

*Affleck, Glenn, et al. "Everyday life with osteoarthritis or rheumatoid arthritis: independent effects of disease and gender on daily pain, mood, and coping." PAIN® 83.3 (1999): 601-609.*

*Kvien, Tore K. "Epidemiology and burden of illness of rheumatoid arthritis." Pharmacoeconomics 22.1 (2004): 1-12.*

*Ahlmén, Monica, et al. "Influence of gender on assessments of disease activity and function in early rheumatoid arthritis in relation to radiographic joint damage." Annals of the rheumatic diseases 69.01 (2010): 230-233.*

(Comment 7) Does the iterative process guarantee the conversion of estimated cell type proportions? The theory behind converging behavior is lacking.

*Response:This is a great question and something we had thought about while developing the method. We first acknowledge that we cannot provide theoretical justification to guarantee the convergence of the estimated proportions. However, our method can guarantee the improvement of proportion estimations. Remember the algorithm iterates between two steps: proportion estimation and feature selection. Thus, the improvements come from two aspects. The first is that RF deconvolution performs better with more “correct” or informative features, and the second is that feature selection can be improved with more accurate estimated proportions. The first point has been empirically demonstrated by all our simulation and real data results, and by discussions about feature selection in many RF deconvolution*

*publications (Rahmani et al., Nature methods, 2016; Rahmani et al., Genome Biology, 2018). The latter point is a natural result from the measurement error model (Gleser, The Annals of Statistics, 1981; Pischke, London School of Economics, 2017; Abel, Technical Report, 2018), which was shown that smaller biases in proportion matrix (V) leads to better statistical inference in selecting cell type specific markers. This is also shown by our simulation results that number of selected true markers is increased after a few iterations.*

*We have added a new paragraph in the Method and material section (page 14 paragraph 1) to discuss these issues.*

*References for Comment 7:*

*Rahmani, Elior, et al. "BayesCCE: a Bayesian framework for estimating cell-type composition from DNA methylation without the need for methylation reference." Genome biology 19.1 (2018): 141.*

*Rahmani, Elior, et al. "Sparse PCA corrects for cell type heterogeneity in epigenome-wide association studies." Nature methods 13.5 (2016): 443.*

*Gleser, Leon Jay. "Estimation in a multivariate" errors in variables" regression model: large sample results." The Annals of Statistics (1981): 24-44.*

*Pischke, Steve. "Lecture notes on measurement error." London School of Economics, London (2007).*

*Abel, Andrew B. Classical measurement error with several regressors. Tech. Rep.). Working Paper, 2017.*

(Comment 8) Whether the same framework could be applied in high-throughput datasets generated by non-microarray-based platforms is unclear. Not addressing this point is a missed opportunity. The authors commented that the algorithm should work with RNA-seq, but they didn't test it due to the lack of good RF deconvolution method. However, some tests could be done focusing entirely on feature selection part (which doesn't require deconvolution) even with just simulated data.

*Response:* *Thank you for the constructive suggestion! We agree that it is important to test our algorithm on RNA-seq data for the feature selection function. To address this point, we designed a simulation study based on real RNA-seq data from GEO (accession number GSE60424). This dataset contains RNA-seq measurements for 6 purified blood cell types, each with 4 replicates for healthy subjects. We simulated a total 100 samples with Bioconductor package PROPER using the parameters estimated from the above real RNA-seq dataset. We manually mixed pure cell type profiles to generate means for mixed samples, and then use negative binomial model to generate observed counts. The details of this simulations study has been added to our Supplementary Material Section S2. With the simulated datasets, we tested our algorithm for detecting cross-cell type differential signals and the results were presented in Figure S17. The proposed algorithm is able to detect cross-cell type differential expressed genes (DEGs) with high accuracy (>70% of the top ranked 1000 genes are true DEGs), which means our proposed algorithm is able to accurately select desired features from RNA-seq data. We have added related discussions to the Discussion section (page 10 paragraph 4).*

**Reviewer 2**

This paper presents TOAST, an iterative method for feature identification for use in reference free cell type decompositions. The idea is to iterate between (a) using features to identify the cell type proportions and (b) updating features corresponding to the cell type proportions. The method is thus compatible with all current reference-free cell type deconvolution methods. The authors have done a nice job of performing the comparison and have made the software available via an R package via Github.

*Response :Thank you for the positive comments!*

Major comments:

(Comment 1) It seems like the approach proposed by the authors is in some respects more similar to methods like RUV or independent SVA - iterated methods that alternate between improving a feature set and calculating the corresponding cell types (or other latent factors). It would be useful to have some discussion or comparison to these methods to see how they are different/similar to the proposed approach. One obvious difference is that those methods can't be used with any other reference free decomposition approach, but this could be noted.

*Response: Thanks for the insightful comment. It is true that both RUV and ISVA have some connections to our proposed method. They are targeting at removal of “unwanted” variations or “undesired” confounding factors. The cell compositions, which we are interested in, might be “unwanted” variations or confounding factor under their context. In addition, all methods use factor analysis: RUV uses SVD, ISVA uses ICA, and TOAST use NMF.*

*There are also important differences among the methods. RUV uses a fixed set of pre-selected features (mostly negative control genes such as housekeeping genes, spike-ins, or ERCC). There is no feature selection, thus RUV is fundamentally different from our method. ISVA has more similarity to our method for its data-driven feature selection procedure, however, there are two important differences. First, ISVA identifies features that are highly correlated with each individual surrogate variable (SV), so it’s based on a simple regression. On the contrary, TOAST selects features based on a statistical test involving several coefficients from a multiple regression. If the SVs contain proportion information, the ISVA approach can be considered as a special case of TOAST. Secondly, ISVA only performs one round of feature selection instead of iterating between feature selection and ICA (Independent Component Analysis). This may not be ideal since we have shown that the iteration greatly improves the results.*

*We have cited all papers and added a paragraph to discuss this issue in the Discussion section (page 11 paragraph 3).*

(Comment 2) It would be useful to have a more thorough evaluation of the sensitivity to the initial feature selection. It is a little uncomfortable that the initial features matter so much. It seems like a more general statement of how to select the initial set of features and how to evaluate whether they are "good" or not is important. Some things I'd like to see are:

- A more thorough evaluation of the sensitivity depending on the method

- Examples from multiple data sets to get some kind of "general" idea about what works or doesn't

- Some suggestions for users that are general purpose in how to select the initial features

- Some discussion of how "off" the results could be for given choices

- Some way to evaluate whether the initial selection was "good" or not

*Response* *Thanks for the comments. These are good points. We conducted additional simulation studies in both gene expression data and DNA methylation data to explore the impact of initial marker selection. We compared the performance of TOAST using different initial markers: the top most variable 1000, 1001-2000, 2001-3000, 5001-6000, 10001-11000 features as well as 1000 randomly selected features. All results are summarized over 100 Monte Carlo datasets. As demonstrated in Figure 3 (previous Figure 3 has been moved to Figure S6) and S7, TOAST is very robust and stable over different selections of initial markers. The improvements from without applying TOAST (Initial column) to after applying TOAST (TOAST column) are consistent and stable across different selections of initial markers (columns) and different sample sizes (rows), and different data types (gene expression data in Figure 3 and DNA methylation in Figure S7).*

*These results show that TOAST is not sensitive to the initial feature selection. As commented in our manuscript “Initial feature selection” section, the only difference is that the top 1000 most variable features make TOAST converge a bit slower than 1001-2000 markers. Still, 30 iterations guarantee satisfactory results for both selections. In addition, we find that even a randomly selected 1000 features work well with TOAST (shown in the last column in the figures below). Considering the differences of outcomes using these initial markers are small, we still use the top 1001-2000 features as the default initial features in our package.*

*We have added related descriptions and discussions in the Background section (page 2 paragraph 2)., “Initial feature selection” (page 4-5)., and Discussion section (page 10 paragraph 3).*

*Gene expression results:*

*DNA methylation results:*

Minor comments:

(Comment 3) The code is available on Github, but it would be much more confidence inspiring if the code was available via Bioconductor after a proper code review

*Response:* *We completely agree with this comment. We actually have submitted our package to Bioconductor right after submitting our manuscript to the editorial office. After two rounds of reviews in a month, our package has been accepted to Bioconductor. Now TOAST is available on Bioconductor at https://bioconductor.org/packages/TOAST. We have updated this information in both main manuscript (page 3 paragraph 1) and “Availability of data and materials” section (page 16).*

(Comment 4) I think the very small p-values are distracting (it doesn't really make sense to report a p-value less than 1x10^-16 or so) the effect size is much more important)

*Response: Thanks for the comment. We have modified all small p-values to p < 1e-16 across all figures (specifically Figure 2(b), 4(a)(c), S1, S2, S3).*

**Reviewer 3**

Li and Wu present TOAST, a computational approach for feature selection to aid deconvolution of omics datasets. The approach is based on a greedy heuristic, at each iteration of which, a subset of original input features used for the deconvolution run is selected based on significance in the linear model fitting the cell type proportion estimates to the observed data. While the idea behind the method appears original and sound, there are several major issues that have to be resolved before the paper can be considered for publication:

Major issues:

(Comment 1) The description of the mathematical methodology is too superficial for a computational "Method" paper, and some crucial technical details are completely missing. For instance, it is not clear which method the authors are using to fit the model described on p.10:51-57, or which statistical procedure they use to test the significance of the null hypothesis defined in line 61, same page (by the way, the number of displayed equations is large enough to introduce numbering for convenience of referencing). To bring it to the level of the present journal, the Methods section have to be substantially revised, and the authors should invest a major effort into clarifying their approach with all necessary details.

*Response Thanks for the helpful suggestions. We have modified our methods section to include all necessary technical details to solve our proposed model. The specific added details include: 1. Our linear model was fit using ordinary least square; 2. We imposed data-driven shrinkage on the solved mean squared error, to stabilize the estimations; 3. We provide options to bound the negative estimated parameters (page 13 paragraph 1). As suggested, we have also numbered the presented equations.*

(Comment 2) Assuming that the linear model is fit using OLS, do the authors perform any feasibility checks on the obtained coefficients, which, according to the authors, should represent mean levels of a feature (gene expression level or DNA methylation level) in a given cell type. In particular, how do the authors deal with negative coefficient values that can arise in the absence of any biologically motivated constraints?

*Thanks for the question.*

*First of all, the negative values only consist a small proportions of total features (~ 1.1% of all estimated parameters in RA data, ~ 2% in simulation data).*

*Second, We could impose constraints on the regression coefficient to avoid negative coefficients. This, however, would greatly undermine the computational performance of the package. We have also tried the most straight-forward method by bounding the negative estimate at 0. This approach is commonly used in other methods with similar purpose. For example, we find the CellDMC paper (Zheng et al., Identification of differentially methylated cell types in epigenome-wide association studies, Nature Methods, 2018) uses exactly the bounding approach to remove negative values in their implementation. However, we find bounding the negative values have minimum impact on the results (see Figures below).*

*Simulation results after bounding the negative values:*

*Real data results after bounding the negative values:*

*Therefore, we believe whether to bound negative values, or which approach to deal with negative coefficient values should have minimum impact on our results. Depending on the preferences of users, we provide options to bound the negative values in our TOAST package.*

*We have added discussions about the bound options in the method section (page 12 paragraph 4).*

(Comment 3) Did the authors make any thought about the possible conversion criteria of their algorithm? Naïve intuition hints that the subset of input features that represent true cell type-specific markers is expected to stabilize with the progression of the iterations, allowing to define a straightforward stopping criterion (for example, less than 5% of subset size change in 5 consecutive iterations).

*Response: Thanks for sharing your thoughts and for the suggestion. Indeed we have thought about the converging behavior of the proposed methods and possible endpoint selection criteria.*

*We first acknowledge that we cannot provide theoretical justification to guarantee the convergence of the estimated proportions. However, our method can guarantee the improvement of proportion estimations. Remember the algorithm iterates between two steps: proportion estimation and feature selection. Thus, the improvements come from two aspects. The first is that RF deconvolution performs better with more “correct” or informative features, and the second is that feature selection can be improved with more accurate estimated proportions. The first point has been empirically demonstrated by all our simulation and real data results, and by discussions about feature selection in many RF deconvolution publications (Rahmani et al., Nature methods, 2016; Rahmani et al., Genome Biology, 2018). The latter point is a natural result from the measurement error model (Gleser, The Annals of Statistics, 1981; Pischke, London School of Economics, 2017; Abel, Technical Report, 2018), which was shown that smaller biases in proportion matrix (V) leads to better statistical inference in selecting cell type specific markers. This is also shown by our simulation results that number of selected true markers is increased after a few iterations. We have added a new paragraph in the Method and material section (page 14, paragraph 1) to discuss these issues.*

*Regarding to endpoint selection, it is possible (and we have observed in previous experiments) that the results stay similar for a consecutive number of iterations (say 5), and then reach a big improvement after updating a new round of features. As a result, we think a stopping criterion based on differences of consecutive iterations may not be a good choice. And we find choosing results by smallest RMSE among 30 (or more) iterations is our current best options. We have justified this choice by Figure 4 and S5.*

*References for Comment 3:*

*Rahmani, Elior, et al. "BayesCCE: a Bayesian framework for estimating cell-type composition from DNA methylation without the need for methylation reference." Genome biology 19.1 (2018): 141.*

*Rahmani, Elior, et al. "Sparse PCA corrects for cell type heterogeneity in epigenome-wide association studies." Nature methods 13.5 (2016): 443.*

*Gleser, Leon Jay. "Estimation in a multivariate" errors in variables" regression model: large sample results." The Annals of Statistics (1981): 24-44.*

*Pischke, Steve. "Lecture notes on measurement error." London School of Economics, London (2007).*

*Abel, Andrew B. Classical measurement error with several regressors. Tech. Rep.). Working Paper, 2017.*

More generally, the final numbers of predictive features are neither presented nor discussed, except for the overlap with the cell type-specific markers based on reference data. Moreover, the latter overlaps could be presented more informatively (e.g., as Venn diagrams) in order to give a more complete picture of what is going on.

*Response* *Thanks for the comment. For every iteration, we select 1000 features based on the cross-cell type differential analysis results. As you suggested, we have investigated the overlaps among initial features, cell type-specific features, and final features. Figure S8 presented such information and we find that the overlaps between final features and initial features are all overlapped with cell type-specific features. And compared with Initial features, final features have ~200 more overlaps with cell type-specific features. We have included related descriptions to the “Initial feature selection section” (page 4 paragraph 2).*

(Comment 4) When the performance after applying TOAST is exceeding that of true cell type-specific markers, what is the nature of features that are not in the true set? Are those also good markers, missed in the analysis of reference data? How high were they ranked in the latter analysis?

*Response: Thank you for the questions. First of all, the differences between the TOAST-selected features and cell type-specific features are caused by the biological variances and biases of the reference panel (pure cell type profile). In real world scenario, reference panels (W) are often obtained from a population different from the samples being studied. Thus, there could be biases in the reference. TOAST obtains cell type-specific features based on the mixed measurements from the current samples. When the biases in the existing reference panels are large, TOAST can achieve better performance than using external reference panel.*

*Yes, the TOAST features that are not in the true set are good markers. They were not ranked at the top in reference, but ranked high in our cell type specific analysis. This is caused by the biases in reference, as the reference can be deviated from the personalized reference panels of the population. As you suggested, we have plotted the rankings of these TOAST-specific markers in reference-data analysis results for all cell types. The figure below shows that the majority of these features ranked within the top 5000 (top 10 percent) cell type-specific features (out of 54675 total features).*

(Comment 5) Which role does genetic variation play in this feature selection problem (QTLs and methQTLs). Which fraction of most variable features can be still attributed to genetic variation? In the case of DNA methylation, will the performance with 1000 most variable features improve after filtering for SNPs-overlapping CpG probes?

*Response:* *Thanks for the question. We didn’t consider genetic variation in this work. It is true that some most variable features can be caused by genetic variation. However, the cell type specific features selected by TOAST should be the ones that vary among different cell types but are relatively constant cross individuals. Since for an individual, different cell types usually share the same genotype, the genetic variation shouldn’t play a role in selecting cell type specific features.*

*On the safer side, one could remove features that are known to be impacted by genetic variations if such information is available. For most real datasets used in this work, the genetic variation information is not available. But as you suggested, we have investigated the influence of removing SNPs-overlapping CpG probes. Among a total of 485577 CpG probes, 401101 were left after removing SNPs-overlapping CpG probes. We analyzed RA data with or without removing SNPs-overlapping CpG probes (Figure below). We found the performance of TOAST does not change significantly. Thus we still stick with our previous results.*

(Comment 6) The authors test first and second 1000 most variable markers both, for expression and DNA methylation data, despite that the number of features on the DNA methylation microarray is an order of magnitude bigger than the number of genes. In the case of larger data sets (e.g. whole-genome DNA methylation profiles), several thousand features might be much too little to pick up essential cell type-attributed variability. In that respect it would be important to know how the algorithms scale in terms of the number of input features.

*Response:* *Thank you for the comment. We agree it is important to evaluate the computational performance of the proposed method. To address this point, we benchmark our proposed method in one simulation dataset based on gene expression data (total number of features = 54,675) or DNA methylation data (total number of features = 459,226). Note that TOAST used the information from all features in the analysis and select a subset of features each round to perform reference-free deconvolution. We examined number of selected features ranges from 100 to 10,000. The table below demonstrates that, TOAST scales well to large datasets. For example, TOAST with 459,226 features with 10,000 features selected in each iteration only takes about 11 mins, and one third of the time is taken by reference-free algorithms.*

*We have included descriptions and discussions about the computing performance in Discussion section (page 11 paragraph 2).*

(Comment 7) Can goodness of fit (p.12,55-62) be used as an adequate evaluation metrics, as it is, indirectly, the selection criterion in the, so called, cross-cell type differential analysis (authors' definition), and is hence bound to increase after the addition of the TOAST method? Rows resulting in a significant fit are automatically leading to a higher correlation coefficient with the observed data.

*Thanks for the comment. In general we agree that different selections of features could impact goodness of fit. However, we are not selecting rows with overall significant fit. Rather, we select the ones that are significant for cell type specific change, which is a one degree of freedom test and not obviously correlated with the overall significance of the linear model. We use goodness of fit to evaluate the results mainly when true proportions or external reference panel are not available (for example, the Breast dataset), in which cases goodness of fit becomes the only viable evaluation approach (RMSE could be calculated but we use RMSE to choose endpoint, which biases the evaluation). A further investigation shows that goodness of fit is positively correlated with ”Corr with True Prop” (Figure below, and now included as Figure S11). Therefore, we believe that the results with high goodness of fit are more likely to be associated with higher correlation with true proportions. This makes the goodness of fit a reasonable criterion to evaluate the results.*

*Moreover, goodness of fit with the exact definitions as ours has been used in other high-profile publications. For example, “Leveraging heterogeneity across multiple datasets increases cell-mixture deconvolution accuracy and reduces biological and technical biases” by Vallania et al. on Nature Communications, 2018 has extensively used goodness of fit to evaluate the quality of deconvolution results.*

*Lastly, goodness of fit is only one of many evaluation criteria used in this work. Other criteria, correlations with true proportions, number of overlaps with cell type specific features, root mean squared error, and root mean squared bias, have also justified the favorable performance of the proposed method. We hope using other criteria is a strong complementary and reassurance to including goodness of fit as a part of the evaluation metrics.*

*We have added related contents about goodness of fit in Real data results (page 7 last paragraph).*

(Comment 8) The ability to predict patients' disease status from estimated proportions (as the authors have done it for the rheumatoid arthritis dataset) is, generally, a bad evaluation criterion for the methods estimating cell type proportions, since there is no guarantee that cell type-independent, yet disease-relevant variation is not translated into less accurate cell type proportion estimates.

*This is a great comment. First of all, we completely agree that better disease prediction does not necessarily link to better proportion estimations, and vice versa. For example, many diseases do not influence cell compositions in any way, and thus disease status is not directly associated with cell type proportions. However, some other diseases are known to change cell compositions in patients compared to controls. For these diseases, better disease prediction is more likely to be associated with better proportion estimation.*

*Rheumatoid arthritis (RA) is such a disease. It has been reported in previous publications that RA can significantly changes the proportions of blood cell types in patients (Hidaka et al., Therapeutic apheresis, 1999; Kikuchi et al., Arthritis research & therapy, 2015), making blood cell proportions from RA patients differ from healthy subjects. Therefore, the blood cell proportions can potentially be used for predicting RA. Based on these information, we believe it is reasonable to conclude that, in our RA study, better predictions is more likely to indicate better proportion estimations. We have include these rationale in the “Comparison of RB and RF estimations in RA data” (page 9 paragraph 1).*

*References for Comment 8:*

*Hidaka, Toshihiko, et al. "Changes in CD4+ T lymphocyte subsets in circulating blood and synovial fluid following filtration leukocytapheresis therapy in patients with rheumatoid arthritis." Therapeutic Apheresis 3.2 (1999): 178-185.*

*Kikuchi, Jun, et al. "Peripheral blood CD4+ CD25+ CD127 low regulatory T cells are significantly increased by tocilizumab treatment in patients with rheumatoid arthritis: increase in regulatory T cells correlates with clinical response." Arthritis research & therapy 17.1 (2015): 10.*

Minor points:

(Comment 9) p.4:48, Wrong figure references (should be Figure 3)

*Thanks for the comment. We have double checked our manuscript, and believe our figure reference here is correct. In this paragraph, we are comparing the converging rate of different initial feature selection (Figure 2, S1, S2, S3 present such results). Figure 3 (now Figure S6) is referenced in the next paragraph to illustrate the possible reasons of different converging rates.*

(Comment 10) p.10:48, "can by modeled" should read "can be modeled"

*Thanks for pointing this out. We have corrected the typo.*

**Second round of review**

**Comments to author and authors’ point-by-point responses to the reviewers’ comments:**

**Reviewer 1**

I thank the authors for their effort in addressing my criticisms. I do not have further comments.

**Reviewer 2**

I believe the authors have addressed the queries I raised on the original review.

**Reviewer 3**

I have now carefully considered the authors' response to my and other reviewers' comments. I found most of my initial issues satisfactory addressed, many thanks, great job! Nevertheless, a couple of minor points would be useful to resolve: Thank you for the positive comment!

(Comment 1) I am still missing some examples of the dynamics of feature size across the iterations. Similar to the improvement curves of "Correlation to True Proportions" vs "#Iterations" it would be interesting to see how the actual feature size changes along the execution of TOAST. Am I getting it correct that ALL significant features based on (3) are selected in each iteration or only a subset of those (little detail about that in the Methods)? This can have dramatic influence upon the performance of the enclosed reference-free deconvolution method.

*Response:* *No, our method does not select all significant features. Instead, we choose the a total of 1000 features based on cross-cell type differential analysis. This is done by taking the top 1000/K features for each cell type, where K is the number of cell types. So if there are 4 cell types, we take 250 most significant features for each cell type. Thus, the number of selected features in each iteration does not vary, but stays the same as the number of initial features. In our Bioconductor package implementation, the function for this utility “DEVarSelect()” has an argument “nMarker”. It specifies the number of initial features as well as the number of selected features in each iteration. The default is 1000, but users can specify other values as they wish. We have added clarifications about number of features selected in each iteration to the Method section second paragraph on page 13 and Algorithm 1 step 5 on page 14.*

(Comment 2) Concerning the influence of genetic variation, in particular the SNP-overlapping and methQTL CpGs, the authors report no significant improvement of TOAST performance when those are removed. It is however interesting which influence they have upon the performance in initial sets. In particular, it was somewhat surprising (or actually not) to see that the initial highly variable sets starting from 5000 most variable CpGs (5001-6000) etc, have much better performance compared to the higher ranking one (see your answers to Reviewer 1 Comment 4 and Reviewer 2 Comment 2). Can it be that this is due to high number of genetically affected CpGs in the higher ranking subsets?

*Response: Thanks for the question. We investigated the number of overlaps between the top variable features, TOAST selected features, and the SNP-overlapping features in the EPIC DNA methylation data. The figure below shows that, no matter where the selected features are located (even the 5001-6000), the number SNP-overlapping CpGs sites is close to the expected value (red bar at the last column). This result indicates that the feature selection is not affected by genetic alterations, regardless of the method. Based on our previous results (Figure S6), we showed that the top variable CpG sites have higher within cell type variations compared to the medium variable ones. We believe this undesirable large within cell type variations, instead of genetic influence, causes the inferior performance for deconvolution in initial run. Nevertheless, the initial feature selection is not the focal point of this paper, since we have shown that the proposed method performs well regardless of the initial feature set.*
